# Molecular Recognition by Zn(II)‐Capped Dynamic Foldamers

**DOI:** 10.1002/open.201900362

**Published:** 2020-03-18

**Authors:** Natasha Eccles, Flavio della Sala, Bryden A. F. Le Bailly, George F. S. Whitehead, Jonathan Clayden, Simon J. Webb

**Affiliations:** ^1^ Department of Chemistry University of Manchester Oxford Road Manchester M13 9PL UK; ^2^ Manchester Institute of Biotechnology University of Manchester 131 Princess St Manchester M1 7DN UK; ^3^ School of Chemistry University of Bristol Cantock's Close Bristol BS8 1TS UK

**Keywords:** peptides, receptors, self-assembly, molecular recognition, supramolecular chemistry

## Abstract

Two α‐aminoisobutyric acid (Aib) foldamers bearing Zn(II)‐chelating N‐termini have been synthesized and compared with a reported Aib foldamer that has a bis(quinolinyl)/mono(pyridyl) cap (BQPA group). Replacement of the quinolinyl arms of the BQPA‐capped foldamer with pyridyl gave a BPPA‐capped foldamer, then further replacement of the linking pyridyl with a 1,2,3‐triazole gave a BPTA‐capped foldamer. Their ability to relay chiral information from carboxylate bound to Zn(II) at the N‐terminus to a glycinamide‐based NMR reporter of conformational preference at the C‐terminus was measured. The importance of the quinolinyl arms became readily apparent, as the foldamers with pyridyl arms were unable to report on the presence of chiral carboxylate in acetonitrile. Low solubility, X‐ray crystallography and ^1^H NMR spectroscopy suggested that interfoldamer interactions inhibited carboxylate binding. However changing solvent to methanol revealed that the end‐to‐end relay of chiral information could be observed for the Zn(II) complex of the BPTA‐capped foldamer at low temperature.

## Graphical abstract

**Crossed wires**: Two foldamers with Zn(II) chelated at their N‐termini have been analyzed for their ability to bind chiral carboxylates and relay this chiral information to a glycinamide reporter at the C‐terminus. X‐ray crystallography and ^1^H NMR spectroscopy suggested that interfoldamer complexation led to poor solubility and inhibited carboxylate binding.

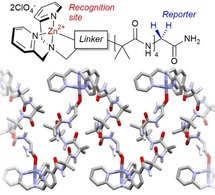

## 1 Introduction

Metal ion complexes of tetradentate tripodal ligands, such as tris(pyrid‐2‐ylmethyl)amine (TPA) and tris(triazol‐2‐ylmethyl)amine, provide versatile scaffolds that have been applied as reversible peroxide binders,^1^ catalysts for phosphodiester cleavage,^2^ fluorescent sensors for a variety of metal ions^3^ and accelerating ligands in Cu(I)‐catalysed azide−alkyne cycloadditions.^4^ In particular, Zn(II) complexes of TPA derivatives have been used to measure the *e.e*. of amino acid^5^ or alcohol mixtures,^6^ while Cu(II) complexes of the closely related ligand *N*,*N*‐bis(quinolin‐2‐ylmethyl)‐*N*‐(pyrid‐2‐ylmethyl)amine (BQPA) were used by Anslyn and Canary to determine the *e.e*. of chiral carboxylic acid mixtures.^7^ This Cu(BQPA)^2+^ complex was reported to adopt two equally populated left‐ (Λ) or right‐ (Δ) handed propeller conformations, with binding of a chiral carboxylate to a vacant coordination site favoring one propeller conformation over the other.

We have used the BQPA motif as a ligand recognition domain on the N‐terminus of a 3_10_ helical α‐aminoisobutyric acid (Aib) foldamer. Metal complexes of these BQPA‐capped Aib foldamers have been used to relay chemical information through the length of Aib foldamers to a reporter group, information that is encoded either in ligand shape (chiral ligands)^8^ or in the *e.e*. of a scalemic mixture of carboxylates.^9^ This relay terminates at a C‐terminal reporter group on the foldamer, such as a bispyrene group,^10^ difluorinated Aib,^11^ or a glycinamide (GlyNH_2_) residue. The latter is a particularly well‐characterized and chemically stable reporter group.^9^, ^12^ The 3_10_ helical conformation of the foldamer renders the GlyNH_2_ methylene protons diastereotopic, but since achiral Aib foldamers exist as a racemic mixture of left‐handed (*M*) and right‐handed (*P*) 3_10_ helices, at fast exchange on the NMR timescale the methylene resonance coalesces to a single 2H signal. The binding of a chiral ligand at the N‐terminus perturbs this *M*/*P* equilibrium, leading to anisochronous methylene resonances; the resulting ABX system has a chemical shift separation (*Δδ*) that is proportional to the excess of one screw sense over the other (the helical excess, *h.e*.).^13^ Although these BQPA‐capped Aib foldamers have proved to be remarkably successful for detecting chiral stimuli,^8^, ^9^ the basic and nucleophilic BQPA headgroup limits subsequent chemical transformations of the C‐terminus. Simpler structural variants of BQPA are of interest, especially if they are more robust and easier to synthesise in better yield. These should behave in the same manner as BQPA, favoring coordination of chiral ligands to a chelated metal ion and facilitating transmission of this chiral signal through the Aib foldamer to the remote C‐terminus.

**Figure 1:**
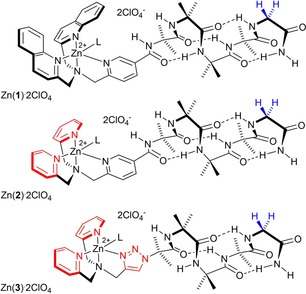
Structures of Zn(**1**) ⋅ 2ClO_4_, Zn(**2**) ⋅ 2ClO_4_ and Zn(**3**) ⋅ 2ClO_4_ with bound ligand L. Structural changes from **1** are shown in red, the methylene protons in the glycinamide are shown in blue.

**Scheme 1:**
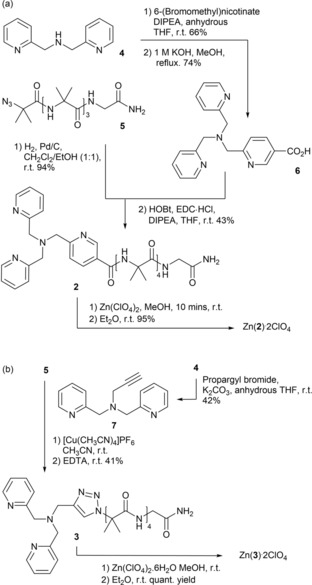
Synthesis of Zn(II) complexes of the Aib foldamers (a) Zn(**2**) ⋅ 2ClO_4_ and (b) Zn(**3**) ⋅ 2ClO_4_, starting from DPA **4** and N_3_Aib_4_GlyNH_2_ **5**.

Using foldamer **1** as the inspiration for our design (Zn(II) complex shown in Figure 1),^9^ two modifications were proposed. The first modification was replacement of the quinolinyl groups with pyridyl, providing analogue **2** that is capped with an *N*,*N*‐bis(pyrid‐2‐ylmethyl)‐*N*‐(5’‐carboxypyrid‐2‐ylmethyl)amine (BPPA) group. This takes advantage of the commercial availability of di‐(2‐picolyl)amine (DPA) **4** and allows a dialkylation step to be replaced with monoalkylation involving **4**. The second modification was replacement of the pyridyl linker with a triazole, providing analogue **3** capped with an *N*,*N*‐bis(pyrid‐2‐ylmethyl)‐*N*‐((1*H*‐1,2,3‐triazol‐4‐yl)methyl)amine (BPTA) group. This modification would produce a different geometry between the foldamer body and the metal chelating group, but gives a product that is available from easily accessible core building blocks such as N_3_Aib_4_GlyNH_2_ **5** (Scheme 1). The BPTA motif is likewise a good binding site for Zn(II).^14^ Building block **5** has a single 3_10_ helical turn, which is in fast exchange between *M* and *P* screw‐sense conformers on the NMR timescale. Anisochronicity in the methylene resonance of the GlyNH_2_ reporter from **5** will form the assay that will report on the performance of the BPPA and BPTA groups within these foldamers.

## 2 Results and Discussion

Foldamer **2** was synthesized in four steps from N_3_Aib_4_GlyNH_2_ **5**, with commercially available di‐(2‐picolyl)amine **4** and 6‐(bromomethyl)nicotinate (Scheme 1). This procedure required two steps fewer than the synthesis of BQPA analogue **1** and removed the need for a double alkylation using 2‐bromomethylquinoline, a step that often gave a hard‐to‐separate monoalkylated byproduct. The overall yield of the BPPA−CO_2_H headgroup **6** from commercially available materials was 49 %, compared to 22 % for the BQPA−CO_2_H headgroup.^15^ The triazole linked analogue **3**, with a BPTA headgroup, was directly synthesized in one step (41 % yield) from N_3_Aib_4_GlyNH_2_ **5** by a copper(I)‐catalysed alkyne−azide cycloaddition (CuAAC) with *N*‐propargyl‐di(2‐picolyl)amine **7**, itself synthesized by alkylation of di(2‐picolyl)amine **4** (42 % yield). Each foldamer was cleanly metallated with Zn(ClO_4_)_2_ to give the corresponding metal complexes Zn(**2**) ⋅ 2ClO_4_ and Zn(**3**) ⋅ 2ClO_4_.

### 2.1 ^1^H NMR Spectroscopy

The chemical shifts of the glycinamide methylene resonances in foldamers **1**, **2** and **3** are typical for glycinamide‐containing foldamers in CDCl_3_,^12^ appearing as singlets at 3.84, 3.75 and 3.91 ppm, respectively. Unlike **1**, complexation to zinc perchlorate sharply reduced the solubility of **2** and **3**; the resulting complexes were soluble or partially soluble in DMSO‐d_6_, CD_3_CN or CD_3_OD.

Foldamer Zn(**2**) ⋅ 2ClO_4_ was much less soluble in CD_3_CN than Zn(**1**) ⋅ 2ClO_4_, and required sonication to dissolve fully. However the ^1^H NMR spectrum of the resulting solution of Zn(**2**) ⋅ 2ClO_4_ in CD_3_CN was sharp with relatively well‐resolved peaks. The methylene protons of the glycinamide at the C‐terminus of this 1 : 1 mixture of rapidly interconverting screw‐sense conformers resonate at 3.72 ppm, appearing as a small doublet due to splitting by the adjacent NH (ESI, Section 2). The same resonance of Zn(**1**) ⋅ 2ClO_4_, also in CD_3_CN, appears at 3.81 ppm. Interestingly, the methylene protons adjacent to the pyridyl groups appeared as singlets, instead of the clear AB system of Zn(**1**) ⋅ 2ClO_4_ (ESI, Section 3.6).

Foldamer Zn(**3**) ⋅ 2ClO_4_ was much less soluble in CD_3_CN than both Zn(**1**) ⋅ 2ClO_4_ and Zn(**2**) ⋅ 2ClO_4_. The ^1^H NMR spectrum of Zn(**3**) ⋅2ClO_4_ in CD_3_CN was broad with poorly resolved peaks (see ESI, Figure S7). Nonetheless it is clear the *M* and *P* conformations are in fast exchange at room temperature, giving a single coalesced 2H glycinamide methylene proton resonance. This was significantly further downfield (at 4.21 ppm, from 3.91 ppm for **3** in CDCl_3_) than in Zn(**1**) ⋅ 2ClO_4_ and Zn(**2**) ⋅ 2ClO_4_, and became concealed by the methylene peaks of the pyridyl and triazole arms (the resonance could be located in the COSY spectrum). This shift, and the broadening of the spectra in CD_3_CN, suggests a potential intermolecular aggregation interaction between the GlyNH_2_ and the electron deficient metal center.

Although still poorly soluble, complex Zn(**3**) ⋅ 2ClO_4_ was more soluble in CD_3_OD than in CD_3_CN and the resulting ^1^H NMR spectrum was simpler (amide NH protons were exchanged) with sharper, defined peaks for all aromatic and alkyl protons (ESI, Section 3.7). Interestingly, this is in contrast to Zn(**1**) ⋅ 2ClO_4_, which had been found to give broader spectra in CD_3_OD than in CD_3_CN, especially in the presence of Boc protected proline (BocPro).^15^ The glycinamide CH_2_ resonance was also shifted upfield from 4.21 to 4.05 ppm, becoming distinct from the pyridyl methylene proton resonances, which may indicate weaker interactions between the GlyNH_2_ and the Zn(BPTA) center in this hydroxylic solvent.^16^ To explore the way inter‐foldamer interactions may be affecting the NMR spectra, a good ligand for Zn(II), chloride, was added to Zn(**3**) ⋅ 2ClO_4_ in CD_3_OD. ^1^H NMR spectroscopy showed the methylene protons of the glycinamide shifted further upfield from *ca*. 4.05 ppm to 3.76 ppm upon chloride addition (ESI, Section 2), which is the usual value for the CH_2_ protons of the GlyNH_2_. The methylene protons of the pyridyl and triazole arms also shifted upfield, also consistent with coordination to a new ligand (chloride) and the loss of any interaction with GlyNH_2_.

### 2.2 Solid State Structures

The X‐ray crystal structure of Zn(**1**) ⋅ 2ClO_4_ has been reported,^9^ so samples of both Zn(**2**) ⋅ 2ClO_4_ and Zn(**3**) ⋅ 2ClO_4_ were subjected to crystallization trials. Although Zn(**3**) ⋅ 2ClO_4_ did not provide good quality crystals, Zn(**2**) ⋅ 2ClO_4_ did provide crystals from methanol that were suitable for structure determination by X‐ray crystallography.

**Figure 2:**
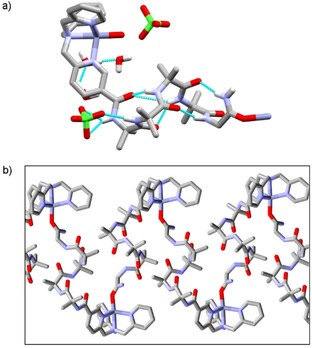
X‐ray crystal structure of Zn(**2**) ⋅ 2ClO_4_. (a) Side view of *M* helix showing the oxygen from the GlyNH_2_ of a neighboring foldamer bound to the Zn(II) ion. Selected hydrogen bonds shown to illustrate the hydrogen bonded network. (b) View showing the glycinamide C=O to Zn(II) interaction that gives head‐to‐tail polymers of *M*‐helical foldamers. Perchlorate counterions, water of solvation and hydrogens are not shown for clarity.

The X‐ray crystal structure of Zn(**2**) ⋅ 2ClO_4_ reveals that, as suspected for Zn(**3**) ⋅ 2ClO_4_, head‐to‐tail aggregation is present, with the C‐terminal glycinamide binding to the Zn(II) center of another foldamer (Figure 2) in the place of solvent coordination (MeOH or H_2_O). This interaction occurs through the carbonyl oxygen of the C‐terminal glycinamide; the C−N bond has a bond length of 1.28(2) Å and the C=O bond has a bond length of 1.27(2) Å, shorter and longer respectively than the same bonds in the uncoordinated glycinamide of Zn(**1**) ⋅ 2ClO_4_ (C−N bond length of 1.319(4) Å, C=O bond length of 1.222(3) Å). These bond length changes are consistent with significant electron density moving onto the oxygen. Aggregation occurred between foldamers of same screw‐sense: both *M* and *P* Aib helical conformations are identified in the unit cell, but the Zn(II) metal center of a *M* helical conformation is found to preferentially interact with the glycinamide of a *M* helix and *vice versa* for *P* helices. The observation of interfoldamer interactions between relatively unfunctionalized foldamers in such a polar solvent^17^ suggests that chiral information could potentially be relayed between appropriately designed foldamers, providing complex systems that sense multiple analytes.^18^

**Figure 3:**
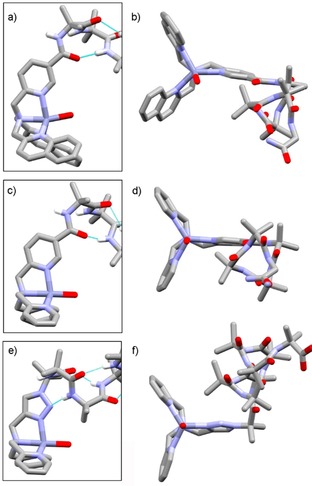
Partial X‐ray crystal structures showing the geometry around the metal center for (a, b) Zn(**1**) ⋅ 2ClO_4_, (c, d) Zn(**2**) ⋅ 2ClO_4_ (glycinamide coordinated in the place of water) and (e, f) Zn(BPTA)(Aib_8_CH_2_CH_2_OSi(CH_3_)_3_) ⋅ 2ClO_4_. Some hydrogens and CH_2_CH_2_Si(CH_3_)_3_ not shown for clarity.^22^

The geometry around Zn(II) in the binding site is consistent with reported [Zn^2+^(TPA)] complexes^19^, ^20^ with near‐ideal trigonal bipyramidal geometry (the Zn(II) lies 0.366 Å above the plane of nitrogens). The Zn−N bond lengths are typical, with an average Zn−PyN bond length of 2.05 Å, and an average propeller twist of 24° (N−Zn−N−C).^21^ Both left‐ (Λ) and right‐handed (Δ) propeller conformations are observed in the unit cell, with a slight Δ propeller conformation found with a *M* helical screw‐sense in the Aib foldamer body whilst the opposite was observed for the Λ propeller conformation (Figure 3d). The Aib foldamer body adopts a distorted 3_10_ helical structure and, as observed in the structure of Zn(**1**) ⋅ 2ClO_4_, a hydrogen bond is present between the carbonyl of the pyridyl linker and Aib_3_ in the 3_10_ helix of the foldamer body (C=O to N(Aib_3_) distance of 3.018(4) Å in Zn(**2**) ⋅ 2ClO_4_, C=O to N(Aib_3_) distance of 3.112(2) Å in Zn(**1**) ⋅ 2ClO_4_).

Interfoldamer complexation to the Zn(II) center in Zn(**1**) ⋅2ClO_4_ is absent, perhaps due to the greater steric demands of the quinolines, which may also produce the more pronounced propeller conformation in Zn(**1**) ⋅ 2ClO_4_ compared to Zn(**2**) ⋅ 2ClO_4_. The axial ligand on the zinc(II) center of Zn(**2**) ⋅ 2ClO_4_ is aligned with the central Zn−N axis, unlike in Zn(**1**) ⋅ 2ClO_4_ where the axial water ligand is out of alignment (Figure 3a,b), which we ascribe to steric clashes with the proton on C8 of the quinolinyl rings.

Although the solid state structure of Zn(**3**) ⋅ 2ClO_4_ could not be determined, the geometry around the Zn(BPTA) headgroup can be inferred from the structure of Zn(BPTA)Aib_8_OCH_2_CH_2_SiMe_3_ (Figure 3e,f).^22^ The Zn(II)(BPTA) group adopts a trigonal bipyramidal geometry (the Zn lies 0.394 Å above the plane of nitrogens in this structure). The pyridyl N to Zn(II) distances (2.04(1) and 2.038(8) Å) are close to those of Zn(**2**) ⋅2ClO_4_ and the triazole N to Zn(II) distance is also similar (2.034(7) Å). The BPTA group has a similar geometry to BPPA, with a slight Λ propeller conformation co‐existing with an *M* helical screw‐sense in the Aib foldamer body (and *vice versa* for the Δ propeller conformation). However, the replacement of the pyridyl group with a triazole results in the 3_10_ helical foldamer body flipping to the other side of the headgroup. An interaction between the headgroup and the foldamer body is evident in the formation of a weak hydrogen bond between N2 of the triazole and the third Aib from the N‐terminus of the 3_10_ helix (N to N(Aib_3_) distance of 3.89(1) Å in Zn(BPTA)Aib_8_OCH_2_CH_2_SiMe_3_ ⋅ 2ClO_4_).^22^ This serves to pull the Aib foldamer body close to the metal complexation site compared to Zn(**2**) ⋅2ClO_4_, although the hydrogen bond is long and its geometry is very distorted.

### 2.3 Effect of Headgroup Structure of the Conformational Relay from N‐ to C‐Terminus

In CD_3_CN, foldamer Zn(**1**) ⋅ 2ClO_4_ can efficiently relay structural information from a bound chiral carboxylate through the helical Aib foldamer body to the C‐terminal GlyNH_2_, where it is revealed in the anisochronicity of the of the GlyNH_2_ methylene resonances. For example, D‐ or L‐BocPro (with 1.2 eq. 2,6‐lutidine, a non‐coordinating base) forms a 1 : 1 complex with Zn(**1**) ⋅ 2ClO_4_ that shows two C‐terminal glycinamide methylene resonances (*Δδ*=181 ppb), shifted slightly upfield to *ca*. 3.66 ppm.^9^

**Figure 4:**
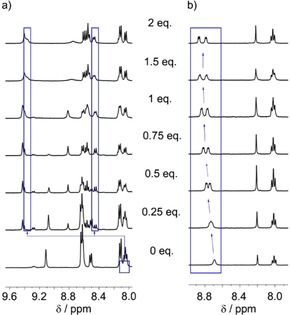
Partial ^1^H NMR spectra showing the aromatic region of (a) Zn(**1**) ⋅ 2ClO_4_ in CD_3_CN and (b) Zn(**3**) ⋅ 2ClO_4_ in CD_3_OD upon the incremental addition of 0 to 2 eq. Boc−D‐Pro (with 0 to 2.4 eq. 2,6‐lutidine). Starting foldamer concentration 0.015 M. The blue boxes show changes in the resonances of (a) protons on the quinolinyl 8‐positions or (b) protons on the pyridyl 2‐positions.

Chemical shift separation between the GlyNH_2_ methylene resonances resulting from induction of a helical screw‐sense preference was also used to monitor the ability of N‐terminal Zn(BPPA) and Zn(BPTA) to relay a chiral signal. Their performance can be compared to the Zn(BQPA) terminus in Zn(**1**) ⋅ 2ClO_4_, which has a number of desirable features. The foldamer with solvent coordinated (“uncomplexed” foldamer) and the foldamer with a chiral carboxylate (typically BocPro) coordinated both give sharp ^1^H NMR spectra in CD_3_CN (although not in CD_3_OD), with these two species in slow exchange (Figure 4a). The interaction between the carboxylate and the Zn(BQPA) in acetonitrile is strong (*K*>10^6^ M^−1^), but interchange between coordinated and uncoordinated carboxylate is fast on the ^1^H NMR timescale.^9^

Upon addition of increasing amounts of Boc−D‐Pro (up to 4 eq., in a 1 : 1.2 ratio with 2,6‐lutidine) to Zn(**2**) ⋅ 2ClO_4_ in CD_3_CN at 25 °C, ^1^H NMR spectroscopy revealed no significant changes in anisochronicity or chemical shifts, even for the amide NH resonances. Nonetheless significant broadening of all resonances from the Aib foldamer body was observed (ESI, Figure S1). Analysis of the analogous titration of Zn(**3**) ⋅ 2ClO_4_ with Boc−D‐Pro was hampered by low foldamer solubility and the glycinamide being obscured under the methylene signals of the BPTA headgroup in this solvent, although once again little binding was apparent. For both complexes it appears that the carboxylate does not coordinate to the Zn(II) center, although conclusive analysis is complicated by spectral broadening.

Changing the solvent from CD_3_CN to CD_3_OD improved solubility and significantly improved the appearance of the ^1^H NMR spectra of Zn(**3**) ⋅ 2ClO_4_; when this foldamer was mixed with Boc−D‐Pro in CD_3_OD this much sharper appearance was maintained. This is also notably different to Zn(**1**) ⋅ 2ClO_4_ in CD_3_OD, which provided broader ^1^H NMR spectra upon Boc−D‐Pro addition. Titration of Zn(**3**) ⋅ 2ClO_4_ with Boc−D‐Pro in CD_3_OD at 25 °C produced anisochronicity (splitting) and an incremental shift in the position of the pyridyl *ortho* proton resonances (Figure 4b), consistent with complexation of the chiral carboxylate to the zinc(II) center. The gradual change in chemical shift shows that, unlike Zn(**1**) ⋅ 2ClO_4_ in CD_3_CN, Zn(**3**) ⋅ 2ClO_4_ in CD_3_OD is in fast exchange on the NMR timescale between uncomplexed (MeOH bound) and complexed foldamer (Boc−D‐Pro bound). This may also indicate weaker binding, and fitting of the data to a 1 : 1 binding isotherm^23^ gave an apparent binding constant of (1.3±0.5)×10^3^ M^−1^ to Zn(**3**) ⋅ 2ClO_4_ in CD_3_OD (ESI, Figure S13), which is around 10^3^ fold weaker than binding of Boc−D‐Pro to Zn(**1**) ⋅ 2ClO_4_ in CD_3_CN. The projected maximum splitting of the pyridyl *ortho* protons (*Δδ*=0.14 ppm) when Zn(**3**) ⋅ 2ClO_4_ is fully bound to Boc−D‐Pro is smaller than the splitting of the comparable protons on the quinolinyl 8‐positions in Zn(**1**) ⋅ 2ClO_4_ (*Δδ*=0.89 ppm) when Boc−D‐Pro is bound.

**Figure 5:**
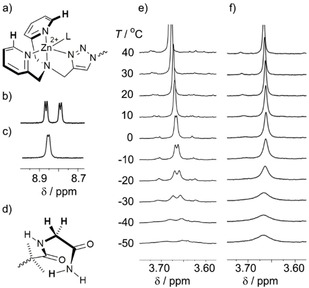
(a) Partial structure showing the protons at the pyridyl 2‐positions of Zn(**3**) ⋅ 2ClO_4_. (b,c) Partial ^1^H NMR spectra showing the resonances from these protons in CD_3_OD after addition of 2 eq. (b) Boc−D‐Pro or (c) *rac*‐BocPro. (d) Partial structure showing methylene protons on the GlyNH_2_ of Zn(**3**) ⋅ 2ClO_4_. (e,f) Partial ^1^H VT NMR spectra from −50 to 40 °C showing these resonances in CD_3_OD after addition of 2 eq. (e) Boc−D‐Pro or (f) *rac*‐BocPro. Each foldamer 0.014 M.

Despite binding of Boc−D‐Pro to the Zn(BPTA) headgroup being apparent at 25 °C (Figure 5b), there was no evidence of stereochemical information being relayed to the Aib peptide and the C‐terminal GlyNH_2_ reporter; anisochronicity at the remote glycinamide methylene and the Aib CH_3_ groups were absent. However decreasing the temperature from 0 °C to −30 °C revealed anisochronicity in the GlyNH_2_ methylene signals. An AB‐system appears at −30 °C with a *Δδ_gly_* of 30 ppb (Figure 5e) and anisochronicity is also observed for the Aib CH_3_ groups at −30 °C with the methyl groups of each Aib residue splitting into two separate signals with very small separation (ESI, Section 4). Decreasing the temperature further to −50 °C caused the broadening of the resonances from the GlyNH_2_ and Aib methyl groups.

To confirm that the anisochronicity in the glycinamide methylene proton resonances arises from a chiral information relay from bound Boc−D‐Pro, the variable temperature NMR (VT‐NMR) spectra of Zn(**3**) ⋅ 2ClO_4_ with 2 eq. of *rac*‐BocPro (2.4 eq. 2,6‐lutidine) were also obtained in CD_3_OD over the same temperature range (Figure 5f). Exchange between unbound BocPro and BocPro bound to the Zn(II) center is fast for *rac*‐BocPro with Zn(**1**) ⋅ 2ClO_4_ in CD_3_CN,^9^ producing averaged isochronous methylene resonances in the GlyNH_2_ of the foldamer. This rapid exchange was also observed for Zn(**3**) ⋅ 2ClO_4_ with *rac*‐BocPro at 20 °C, which showed no anisochronicity in the aromatic resonances from the pyridyl arms of the Zn(II) site (Figure 5c), unlike the mixture with Boc−D‐Pro (Figure 5b). Similarly, no anisochronicity was observed in the GlyNH_2_ methylene resonance at 20 °C. Decreasing the temperature to −50 °C showed the glycinamide (Figure 5f) and Aib methyl group resonances remained unsplit, consistent with the achiral averaged environment arising from rapid exchange of a racemic ligand at the N‐terminus.

## 3 Conclusions

These studies have revealed that headgroup structure strongly influences the ability of these Zn(II) chelating headgroups to relay chiral information from a bound carboxylate down the Aib foldamer to the ^1^H NMR reporter. This relay is not only dependent on the strength or weakness of intramolecular interactions between the foldamer body and carboxylates bound to Zn(II), but measuring the signal from the GlyNH_2_ reporter depends on the way different headgroups favour or disfavour interfoldamer interactions and alter foldamer solubility. The solid state structure of the BPPA‐capped foldamer Zn(**2**) ⋅ 2ClO_4_ revealed an interfoldamer C=O⋅⋅⋅Zn(II) complexation interaction that was not present in the equivalent solid state structure of the BQPA‐capped foldamer Zn(**1**) ⋅ 2ClO_4_; this interaction may indicate why the BPPA‐capped foldamer did not bind Boc−D‐Pro in CD_3_CN. These studies show that a Zn(II) center can drive foldamer self‐association in the absence of a steric shield around the Zn(II) center or the absence of a good ligand in the solution.

A further replacement of the pyridyl group that links the Aib foldamer body to the Zn(II) chelating headgroup with a triazole did not improve the ability of the foldamer to provide an NMR signal upon Boc−D‐Pro addition in CD_3_CN. However changing to CD_3_OD showed Boc−D‐Pro now bound to the Zn(II) in this solvent. Bound and unbound Zn(**3**) ⋅ 2ClO_4_ were in fast exchange, unlike Zn(**1**) ⋅ 2ClO_4_ with Boc−D‐Pro, with the Boc−D‐Pro relatively weakly bound (*K*_app_=10^3^ M^−1^) and producing a small anisochronicity in the Zn(II) chelating arms. At room temperature there was no measurable relay of chiral information to the C‐terminal glycinamide, implying that at 25 °C the carboxylate does not transmit its chirality and both *M* and *P* helical conformations are still equally populated. However lowering the temperature to −30 °C resulted in the observation of weak anisochronicity (*Δδ*=30 ppb) in the methylene signals of the GlyNH_2_, perhaps suggesting key supramolecular interactions between the N‐terminus and foldamer body were better defined at low temperature.

Although the quinolinyl groups in BQPA appear to provide steric bulk that weakens self‐association of foldamer Zn(**1**) ⋅ 2ClO_4_, this in itself will not always provide a suitable foldamer. The analogue of Zn(**3**) ⋅ 2ClO_4_ with quinolinyl groups in the place of the pyridyls also displayed very low solubility in most solvents, including methanol,^15^ indicting the open geometry around the triazole linker promotes self‐association. This example illustrates the often unpredictable way that the solvent affects the ability of these foldamers to give an NMR report. Nonetheless, these studies show that replacing selected coordinating motifs with triazoles can maintain strong chelation of metal ions like Zn(II). Furthermore the triazole linker could also provide a synthetically straightforward means to install at the N‐terminus other recognition groups better able to communicate with the Aib foldamer body.

## Experimental Section

All reactions were carried out in oven‐dried glassware under an atmosphere of nitrogen using standard anhydrous techniques. All reagents were obtained from commercially available sources and used without further purification, or where indicated prepared internally. The synthesis of N_3_Aib_4_GlyNH_2_ **5** and NH_2_Aib_4_GlyNH_2_ have been reported previously.^24^ *N*,*N*‐Di(2‐picolinyl)‐*N*‐(5‐(carboxy)‐2‐picolinyl)amine **6** was synthesized by an adapted method of Hambley and co‐workers (see the ESI).^25^ *N*,*N*‐Di(2‐picolyl)propargylamine **7** was synthesized by a method developed by Zhu and co‐workers (see the ESI).^26^ All products were dried on a rotary evaporator followed by connection to a high vacuum system to remove any residual solvent. Flash chromatography was performed on silica gel (Merck 60H, 40–60 nm, 230–300 mesh) or alumina (Merck, activated, neutral, Brockmann I). Analytical thin layer chromatography (TLC) was performed on Macherey Nagel alugram SIL G/UV254 or TLC Aluminium oxide 60 F_254_, neutral plates and were visualized by UV (254 nm), ninhydrin or potassium permanganate dyes where appropriate.

**General Procedure for the Formation of Zn(II) Complexes**: The ligand (1 eq.) was dissolved in MeOH (20 mL/mmol) and the zinc(II) perchlorate salt (1 eq.) was added dropwise as a solution in MeOH (10 mL/mmol). After 10 min, Et_2_O (90 mL/mmol) was added and the reaction mixture stirred for 1 h. The reaction mixture was filtered and the precipitate washed with Et_2_O to give the complex.

**Foldamer 2**: A stirred solution of **6** (39 mg, 0.116 mmol), NH_2_Aib_4_GlyNH_2_ (65 mg, 0.175 mmol) and HOBt (23.6 mg, 0.175 mmol) in anhydrous DMF (4 mL) were cooled to 0 °C. EDC⋅HCl (31.0 mg, 0.162 mmol) was added in one portion and the reaction mixture was stirred for 10 mins at 0 °C. Following the dropwise addition of DIPEA (60.6 μL, 0.348 mmol), the reaction mixture was warmed to RT and stirred for 2 d. The reaction mixture was concentrated *in vacuo* and re‐dissolved in EtOAc (5 mL) before being washed with sat. NaHCO_3_ (2.5 mL) and brine (2.5 mL). The organic layers were combined, dried (Na_2_CO_3_) and concentrated *in vacuo* to give a yellow residue. Purification by HPLC (Eclipse XD8‐C18, 5 μm, 9.4×250 mm, MeCN:H_2_O 5–51 %) gave the titled compound as a colourless gum (36.5 mg, 43 %). ^1^H NMR (400 MHz, CDCl_3_): δ_H_ 8.99 (1H, br s, Py'H), 8.46 (2H, d, *J=*4.8, 2×PyH), 8.15 (1H, dd, *J=*8.2, 2.1, Py'H), 7.98 (1H, t, *J=*6.4, NHCH_2_), 7.90 (1H, s, NH), 7.83 (1H, s, NH), 7.71–7.64 (2H, m, Py'H, NH), 7.59 (2H, *app* td, *J=*8.0, 1.7, 2×PyH), 7.48 (2H, d, *J=*8.0, 2×PyH), 7.45 (1H, s, NH), 7.12–7.06 (2H, m, 2×PyH), 6.99 (1H, s, NH), 5.32 (1H, s, NH), 3.87 (2H, s, PyCH_2_), 3.80 (4H, s, 2×PyCH_2_), 3.75 (2H, d, *J=*6.2, CH_2_NH), 1.51 (6H, s, 2×CH_3_), 1.47 (6H, s, 2×CH_3_), (6H, s, 2×CH_3_), 1.34 (6H, s, 2×CH_3_) ppm. ^13^C NMR (101 MHz, CDCl_3_): δ_C_ 176.3, 176.2, 175.6, 174.5, 173.4, 166.5, 159.0, 158.8, 149.2, 148.5, 136.7, 135.9, 127.4, 123.1, 122.7, 122.3, 60.0, 56.9, 57.5, 57.0, 56.9, 56.7, 43.2, 25.3, 25.0–24.9 (3×CH_3_) ppm. MS (ES, MeOH): 729.5 (100 %, [M−H]^+^). HRMS (ES, MeOH): *m/z* calcd. for C_37_H_49_N_10_O_6_K [M+K]^+^=769.3551, found 769.3520.

**Zn(2) ⋅ 2ClO_4_**_:_ Following the general procedure, Zn(**2**) 2ClO_4_ (0.015 mmol scale) was afforded as a pale‐yellow solid (23.8 mg, 95 %). ^1^H NMR (400 MHz: CD_3_CN): δ_H_ 9.06 (1H, br s, PyH), 8.72 (2H, d, *J=*5.4, 2×PyH), 8.48 (1H, dd, *J=*8.2, 2.0, PyH), 8.05 (2H, *app* td, *J=*8.0, 1.6, 2×PyH), 7.87–7.84 (2H, m, CONH_2_), 7.63 (2H, m, 2×PyH), 7.61 (1H, m, PyH), 7.58 (2H, m, 2×NH), 7.53 (2H, d, *J=*8.0, 2×PyH), 7.40 (1H, s, NH), 7.20 (1H, s, NH), 6.20 (1H, s, NH), 4.27 (4H, s, PyCH_2_), 4.21 (2H, s, PyCH_2_), 3.72 (2H, d, *J=*6.3, CH_2_NH), 1.48 (6H, s, CH_3_), 1.38 (6H, s, CH_3_), 1.35 (6H, s, CH_3_), 1.28 (6H, s, CH_3_) ppm. ^13^C NMR (101 MHz, CD_3_CN): δ_C_ 177.1, 176.8, 176.41, 176.40, 175.1, 164.5, 158.7, 155.6, 149.1, 148.5, 142.6, 141.5, 131.6, 126.0, 125.6, 125.4, 58.2, 57.7, 57.6, 57.3, 57.2 (2×^α^C), 43.3, 25.3, 25.1, 24.6 (2×CH_3_) ppm. MS (ES, MeOH): 397.2 ([^64^Zn ⋅ **2**]^2+^, 100 %), 795.3 ([^64^Zn ⋅ **2**+H]^+^, 45 %), 832.3 ([^68^Zn ⋅ **2**+MeOH+H]^+^, 45 %). HRMS (ES, MeOH): calc'd for C_37_N_50_O_6_N^64^Zn [M+H]^+^: 794.3206 *m/z* found 794.3203.

**Foldamer 3**: Under an argon atmosphere, N_3_Aib_4_GlyNH_2_ **5** (100 mg, 0.23 mmol) and compound **7** (53.9 mg, 0.23 mmol) were dissolved in dry CH_3_CN (4.5 mL) and the mixture was degassed for 30 min. Then, [Cu(CH_3_CN)_4_]PF_6_ (84.6 mg, 0.23 mmol) was added in one portion and the mixture was stirred overnight at room temperature. The solvent was then evaporated, and the residue was re‐dissolved in CH_2_Cl_2_ (40 mL). The organic phase was washed with sat. EDTA solution (2 mL). The aqueous solution was re‐extracted with CH_2_Cl_2_ (2×5 mL). The combined organic layers were dried over MgSO_4_, filtered and evaporated under reduced pressure. The crude was purified by chromatography (Al_2_O_3_, CH_2_Cl_2_/CH_3_CN/MeOH 5 : 5 : 1 to 0:0 : 1) to yield the title compound as brown oil (63.7 mg, 41 %). ^1^H NMR (400 MHz, CDCl_3_): δ_H_ 8.54–8.53 (2H, dd, *J=*4.9, 0.8, 2×PyH), 7.85 (1H, s, CH), 7.78 (1H, t, *J=*7.7, NHCH_2_), 7.62 (2H, *app* td, *J=*7.7, 1.8, 2×PyH), 7.45 (2H, d, *J=*7.7, 2×PyH), 7.35 (1H, s, NH), 7.28 (1H, s, NH), 7.21 (1H, s, NH), 7.13 (2H, ddd, *J=*7.5, 4.9, 1.2, 2×PyH), 6.72 (1H, s, NH), 5.40 (1H, s, NH), 3.93 (2H, s, TriazoleCH_2_), 3.91 (2H, d, *J=*6.4, CH_2_NH), 3.87 (4H, s, PyCH_2_), 1.86 (6H, s, 2×CH_3_), 1.50 (6H, s, 2×CH_3_), 1.46 (6H, s, 2×CH_3_), 1.39 (6H, s, 2×CH_3_) ppm. ^13^C NMR (101 MHz, CDCl_3_): δ_C_ 175.9, 175.6, 175.0, 173.5, 171.8, 158.5, 149.4, 144.2, 137.0, 123.3, 122.6, 121.9, 65.2, 59.5, 57.6, 57.1, 57.0, 48.8, 43.3, 26.1, 25.3, 24.9, 24.6. MS (ES, MeOH) 678.5 (100 %, [M+H]^+^). HRMS (ES, MeOH): *m/z* calcd. for C_33_H_47_O_5_N_11_ [M+H]^+^=678.3839, found 678.3841.

**Zn(3) ⋅ 2ClO_4_**: Following the general procedure, Zn(**3**) ⋅ 2ClO_4_ was afforded from foldamer **3** (27.4 mg, 0.04 mmol) as a pale‐yellow solid (38 mg, quant). m.p.: <202 °C. ^1^H NMR (400 MHz, CD_3_OD): δ_H_ 8.80 (2H, br d, *J=*5.4, 2×PyH), 8.31 (1H, s, C=CH), 8.13 (2H, *app* td, *J=*7.8, 1.6, 2×PyH), 7.83 (1H, t, *J=*6.2, NHCH_2_), 7.68–7.64 (4H, m, 4×PyH), 7.51 (1H, s, NH), 4.49–4.36 (4H, m, 2×PyCH_2_), 4.29 (2H, s, TriazoleCH_2_), 4.05 (2H, d, *J=*5.6, CH_2_NH), 1.90 (6H, s, 2×CH_3_), 1.45 (6H, s, 2×CH_3_), 1.41 (6H, s, 2×CH_3_), 1.28 (6H, s, 2×CH_3_) ppm. ^13^C NMR (101 MHz, DMSO‐*d*_6_): δ_C_ 174.8, 174.5, 171.4, 170.8, 154.4, 147.6, 141.3, 140.3, 125.0, 124.7, 124.0, 75.4, 66.2, 58.3, 56.8, 56.14, 55.9, 49.9, 42.7, 24.9, 24.8, 24.7, 24.2. MS (ES, MeOH): *m/z* 370.9 ([^64^Zn ⋅ **3**], 90 %), 742.4 ([^64^Zn ⋅ **3**+H]^+^, 100 %), 776.4 ([^66^Zn ⋅ **3**+MeOH+H]^+^, 40 %). IR (ATR, ν_max_): 3351, 1686, 1661, 1614, 1552, 1521, 1439 cm^−1^_._ HRMS (ES, MeOH): *m/z* calcd. for C_33_H_47_O_5_N_11_Zn [M+Zn]^2+^=370.6521, found 370.6507.

**Zn(3).2Cl.Et_2_O**: Foldamer **3** (33.8 mg, 0.05 mmol) was dissolved in MeOH (1 mL) and zinc chloride (6.81 mg, 0.05 mmol) was added dropwise as a solution in MeOH (500 μL). After 10 min, Et_2_O (3 mL) was added and the reaction mixture stirred for 1 h. The reaction mixture was filtered and the precipitate washed with Et_2_O to yield the titled compound as a pale‐yellow solid (46.5 mg, 96 %). ^1^H NMR (400 MHz, CD_3_OD): δ_H_ 9.08 (2H, dd, *J=*5.4, 1.7, 2×PyH), 8.37 (1H, s, C=CH), 8.13 (2H, *app* td, *J=*7.8, 1.7, 2×PyH), 7.84–7.52 (4H, m, 4×PyH), 4.29 (2H, d, *J=*16.8, H^A^ of AB system, PyCH_2_), 4.27 (2H, d, *J=*16.8, H^B^ of AB system, PyCH_2_), 4.17 (1H, s, TriazoleCH_2_), 3.78 (2H, s, CH_2_NH), 1.97 (6H, s, 2×CH_3_), 1.44 (6H, s, CH_3_), 1.37 (6H, s, 2×CH_3_), 1.13 (6H, s, 2×CH_3_) ppm. ^13^C NMR (101 MHz, CD_3_OD): δ_C_ 177.7, 177.3, 176.6, 175.3, 172.4, 156.8, 150.3, 145.0, 142.9, 126.3, 126.2, 124.7, 68.4, 58.5, 58.1, 58.0, 57.8, 57.1, 43.7, 25.7, 25.3, 25.1, 24.6 ppm. MS (ES, MeOH): *m/z* 371.6 ([^64^Zn ⋅ **3**]^2+^, 70 %), 777.3 ([^64^Zn ⋅ **3** +Cl+H]^+^, 80 %), 779.3 ([^64^Zn ⋅ **3**+Cl+H]^+^, 75 %). HRMS (ES, MeOH): *m/z* calcd. for C_33_H_48_N_11_O_5_ZnCl [M+H]^+^=777.2700, found 777.2690.

## Supporting information

As a service to our authors and readers, this journal provides supporting information supplied by the authors. Such materials are peer reviewed and may be re‐organized for online delivery, but are not copy‐edited or typeset. Technical support issues arising from supporting information (other than missing files) should be addressed to the authors.

Supplementary
