# Supplementary material for: Molecular Recognition by Zn(II)‐Capped Dynamic Foldamers
Source: ChemistryOpen. 2020 Mar 18;9(3):338–45. doi: 10.1002/open.201900362 (PMC7080544; doi:10.1002/open.201900362)
Supplement: Supplementary file 1 — Supplementary [file OPEN-9-338-s001.pdf]

Open Access
ChemistryOPEN

Supporting Information

Molecular Recognition by Zn(II)-Capped Dynamic Foldamers

Natasha Eccles, Flavio della Sala, Bryden A. F. Le Bailly, George F. S. Whitehead, Jonathan Clayden,* and Simon J. Webb*© 2020 The Authors. Published by Wiley-VCH Verlag GmbH & Co. KGaA. This is an open access article under the terms of the Creative Commons Attribution License, which permits use, distribution and reproduction in any medium, provided the original work is properly cited.An invited contribution to a Special Collection dedicated to Functional Supramolecular Systems

TABLE OF CONTENTS

1. *Chemical synthesis: materials* 2

1.1 Abbreviations 2

1.2 Instruments 2

1.3 Materials 3

1.4 Modifications of published synthetic procedures 3

N,*N*-Di(2-picolinyl)-*N*-(5-(methoxycarbonyl)-2-picolinyl)amine (**S1**) 3

N,*N*-Di(2-picolinyl)-*N*-(5-(carboxy)-2-picolinyl)amine (**6**) 3

2. *^{1}H and ^{13}C NMR spectra of new compounds* 5

^{1}H NMR of Foldamer **2** in $CDCl_3$ 5

^{13}C NMR of Foldamer **2** in $CDCl_3$ 5

^{1}H NMR of Zn(**2**)·$2ClO_4$·Et_2O in CD_3CN 6

^{13}C NMR of Zn(**2**)·$2ClO_4$·Et_2O in CD_3CN 6

^{1}H NMR spectrum of Foldamer **3** in $CDCl_3$ 7

^{13}C NMR spectrum of Foldamer **3** in $CDCl_3$ 7

^{1}H NMR spectrum of Zn(**3**)·$2ClO_4$ in CD_3OD 8

^{13}C NMR spectrum of Zn(**3**)·$2ClO_4$ in DMSO-d_6 8

^{1}H NMR spectrum of Zn(**3**)·2Cl·Et_2O in CD_3OD 9

^{13}C NMR spectrum of Zn(**3**)·2Cl·Et_2O in CD_3OD 9

3. *NMR spectroscopy studies* 10

3.1 NMR spectroscopy experimental details 10

3.2 Complexation study: titration of Zn(2)·$2ClO_4$ with Boc-D-Pro, 2,6 lutidine in CD_3CN 10

3.3 Complexation study: titration of Zn(3)·$2ClO_4$ with Boc-D-Pro, 2,6 lutidine in CD_3CN 12

3.4 Complexation study: titration of Zn(3)·$2ClO_4$ with Boc-D-Pro, 2,6 lutidine in CD_3OD 14

3.5 ^{1}H NMR spectroscopic comparison of Zn(3)·$2ClO_4$ and Zn(3)·2Cl 16

3.6 ^{1}H NMR spectroscopic comparison of Zn(1)·$2ClO_4$ and Zn(2)·$2ClO_4$ 17

3.7 ^{1}H NMR spectroscopic comparison of Zn(3)·$2ClO_4$ in CD_3OD and CD_3CN 18

4. *Variable Temperature NMR (VT-NMR) spectra of Zn(3)·$2ClO_4$* 19

4.1 VT-NMR spectra of Zn(3)·$2ClO_4$ (either Boc-D-Pro or *rac*-BocPro bound) in CD_3OD 19

5. *Fitting of titration data* 23

6. *Crystal data and structure refinement for Zn(2)·$2ClO_4$* 24

1. Chemical synthesis: materials

1.1 Abbreviations

EDC·HCl = 1-ethyl-3-(3-dimethylaminopropyl)carbodiimide, eq.= equivalents, ES = electronspray, Et_2O = diethylether, BPPA = *N,N*-bis(pyridin-2-ylmethyl)-N-((5'-carboxypyridyl)methyl)amine, BQPA = *N,N*-bis[2-quinolyl]methyl-*N*-[2-pyridyl]methylamine, d = doublet, DIPEA = *N,N*-diisopropylethylamine, DMAP = 2-(dimethylamino)pyridine, Et = ethyl, HRMS: high resolution mass spectrometry, IR = Infra-red, TFA = trifluoroacetic acid, IPA = *iso*propyl alcohol, Hex = hexane, m = multiplet, Me = methyl, MeCN = acetonitrile, MeOH = methanol, mp = melting point, NMR = nuclear magnetic resonance, PE = petroleum ether, Py = pyridine Quin = quinolone, RT = room temperature, s = singlet, THF = tetrahydrofuran, TLC = thin layer chromatography.

1.2 Instruments

All ^{1}H and ^{13}C nuclear magnetic resonance (NMR) spectra were obtained using Bruker AVANCE 400 or 500 spectrometers. Chemical shifts are quoted in parts per million (ppm) and coupling constants (*J*) are quoted in Hz to the nearest 0.5 Hz. ^{1}H NMR shifts were referenced to the solvent peak ($CDCl_3$ 7.27; CD_3OD 3.31; CD_3CN 1.94 ppm) and ^{13}C NMR were referenced to the carbon resonance of the solvent ($CDCl_3$ 77.0; CD_3OD 49.0; CD_3CN 118.2 ppm). Multiplicities are denoted as s (singlet), d (doublet), t (triplet), q (quartet), m (multiplet) or denoted as br (broad), or some combination of these, where appropriate. Assignments were made using 2D ^{1}H-COSY, TOCSY and HMQC experiments. Where ^{1}H NMR spectra were run in CD_3OD, D_2O exchangeable protons (NH, OH) are reported only where observed. The anisochronicity, in parts per billion (ppb), of AB systems arising from the germinal ^{1}H nuclei of the $GlyNH_2$ diastereotopic NMR probe was given by = $[(f1- f3)^2 – JAB^2]^{1/2}$ = $[(f2- f4)^2 -JAB^2]^{1/2}$ = $[(f1- f4)\ (f2- f3)]^{1/2}$ where *f*1,2,3,4 are the observed resonant frequencies in order of the four lines comprising the AB multiplet, J_{AB} is the coupling constant and is the spectrometer frequency.

Melting points (mp) were determined on a GallenKamp apparatus and are uncorrected. Infra-red spectra (IR) were recorded on an ATi Perkin Elmer Spectrum RX1 FT-IR. Only absorption maxima (v_{max}) of interest are reported and quoted in wavenumbers (cm^{-1}). Low- and high-resolution mass spectra were recorded by staff at the University of Manchester. Electrospray (ES) spectra were recorded on a Waters Platform II. High-resolution mass spectra (HRMS) were recorded on a Thermo Finnigan MAT95XP and are accurate to ±0.001 Da. HPLC analyses were performed on an Agilent 1100 Series instrument equipped with an Eclipse XD8- C18 column (5 µm, 9.4 × 250 mm) using different $MeCN:H_2O$ gradients.

1.3 Materials

All reactions were carried out in oven-dried glassware under an atmosphere of nitrogen using standard anhydrous techniques. All reagents were obtained from commercially available sources and used without further purification, or where indicated prepared internally. All products were dried on a rotary evaporator followed by connection to a high vacuum system to remove any residual solvent. Flash chromatography was performed on silica gel (Merck 60H, 40-60 nm, 230–300 mesh) or alumina (Merck, activated, neutral, Brockmann I). Analytical thin layer chromatography (TLC) was performed on Macherey Nagel alugram SIL G/UV254 or TLC Aluminium oxide 60 F_{254}, neutral plates and were visualised by UV (254 nm), ninhydrin or potassium permanganate dyes where appropriate.

1.4 Modifications of published synthetic procedures

N,N-Di(2-picolinyl)-N-(5-(methoxycarbonyl)-2-picolinyl)amine (S1)

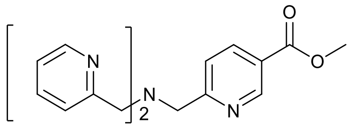

N,N-Di(2-picolinyl)-N-(5-(methoxycarbonyl)-2-picolinyl)amine was synthesised by an adapted method of Hambley and co-workers.[1] Methyl (6-bromomethyl)nicotinate (347 mg, 1.51 mmol) was added to a stirred solution of dipicolylamine (300 mg, 1.51 mmol), DIPEA (785 µL, 4.52 mmol) and anhydrous tetrahydrofuran (10 mL) under argon. The suspension was stirred at RT for 3 d, filtered, and concentrated *in vacuo* to give an orange residue. This was purified by column chromatography (Al_2O_3, EtOAc:PE 1:4 to 1:1) to yield the titled compound as a pale-yellow solid (346 mg, 66%). Spectral data are in accordance with the literature.[1] ***Rf*** (ethyl acetate:PE 2:1) = 0.12. **^{1}H NMR** (400 MHz, CD_3OD): δ_H 9.1 (1H, dd, *J* = 2.2, 0.9, PyH), 8.53 (2H, ddd, *J* = 4.9, 1.8, 0.9, 2 × PyH), 8.24 (1H, dd, *J* = 8.2, 2.2, PyH), 7.69 (1H, dd, *J* = 8.2, 0.9, PyH), 7.65 (2H, *app* td, *J* = 7.5, 1.8, 2 × PyH), 7.54 (2H, d, *J* = 7.9, 2 × PyH), 7.14 (2H, ddd, *J* = 7.5, 4.9, 1.3, 2 × PyH), 3.94 (2H, s, $PyCH_2$), 3.92 (3H, s, OCH_3), 3.89 (4H, s, 2 × $PyCH_2$) ppm. **^{13}C NMR** (101 MHz, $CDCl_3$): δ_C 165.9 (CO), 164.3 (PyC), 159.0 (PyC), 150.3 (PyCH), 149.2 (PyCH), 137.5 (PyCH), 136.5 (PyCH), 124.4 (PyC), 123.0 (PyCH), 122.4 (PyCH), 122.1 (PyCH), 60.3 ($PyCH_2$), 60.0 (OCH_3), 52.3 ($PyCH_2$) ppm. NMR data agreed with reported data.[1]

N,N-Di(2-picolinyl)-N-(5-(carboxy)-2-picolinyl)amine (6)

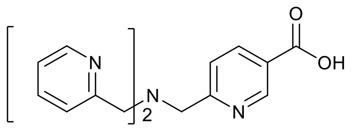

N,*N*-Di(2-picolinyl)-N-(5-(carboxy)-2-picolinyl)amine was synthesised by an adapted method of Hambley and co-workers.[1] Compound **S1** (100 mg, 0.29 mmol) was dissolved in MeOH (9.2 mL) and a 1 M solution of KOH (1.7 mL) was added dropwise. The reaction mixture was heated to reflux and stirred for 2 h. MeOH was

removed and the residue was neutralised using HCl (1 M). The solution was evaporated to dryness and re-dissolved in methanol (5 mL). The insoluble salt was filtered and the filtrate evaporated to give the product as a pale-yellow gum (71.6 mg, 74%). **^{1}H NMR** (400 MHz, CD_3OD): δ_H 8.99 (1H, dd, *J* = 2.1, 0.8, Py'H), 8.35 (2H, ddd, *J* = 5.3, 1.7, 0.8, 2 × PyH), 8.18 (1H, dd, *J* = 8.1, 2.1, PyH), 7.94 (2H, *app* td, *J* = 7.8, 1.7, 2 × PyH), 7.53 (1H, *app* dt, *J* = 8.1, 0.8, PyH), 7.45 (2H, d, *J* = 7.8, 5.3, 1.2, 2 × PyH) 7.42 (2H, dd, *J* = 8.1, 0.8, 2 × PyH), 3.84 (4H, s, 2 × CH_2), 3.81 (2H, s, CH_2) ppm. **^{13}C NMR** (101 MHz, CD_3OD): δ_C 168.7 (CO), 162.7 (PyC), 158.0 (PyC), 151.6 (PyCH), 149.3 (PyCH), 139.9 (PyCH), 139.8 (PyCH), 127.4 (PyC), 125.2 (PyCH), 124.7 (PyCH), 124.4 (PyCH), 60.8 ($PyCH_2$), 60.6 ($PyCH_2$) ppm. NMR data agreed with reported data.[1]

2. ^{1}H and ^{13}C NMR spectra of new compounds

^{1}H NMR of Foldamer 2 in $CDCl_3$

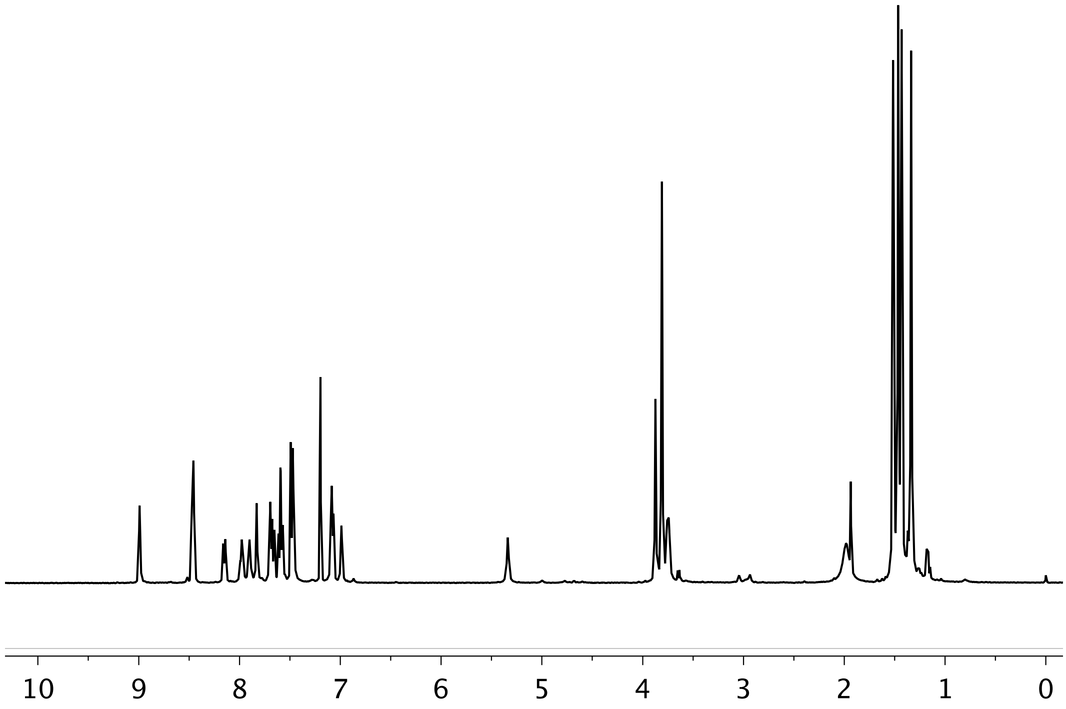

^{13}C NMR of Foldamer 2 in $CDCl_3$

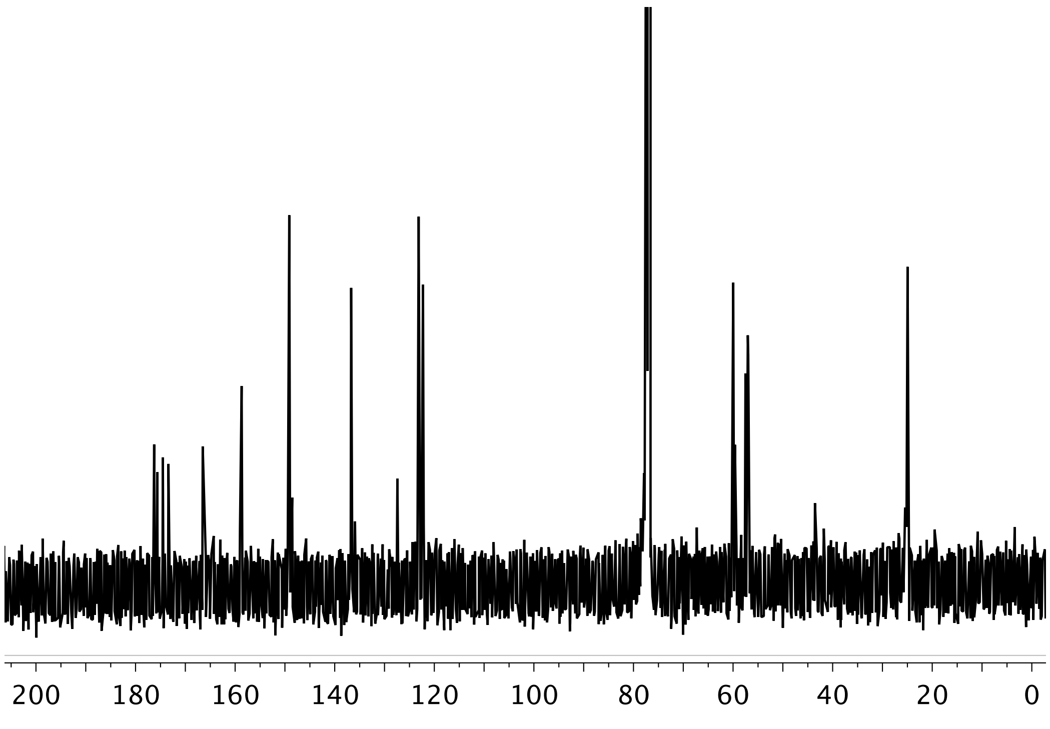

^{1}H NMR of Zn(2)·2ClO_4·Et_2O in CD_3CN

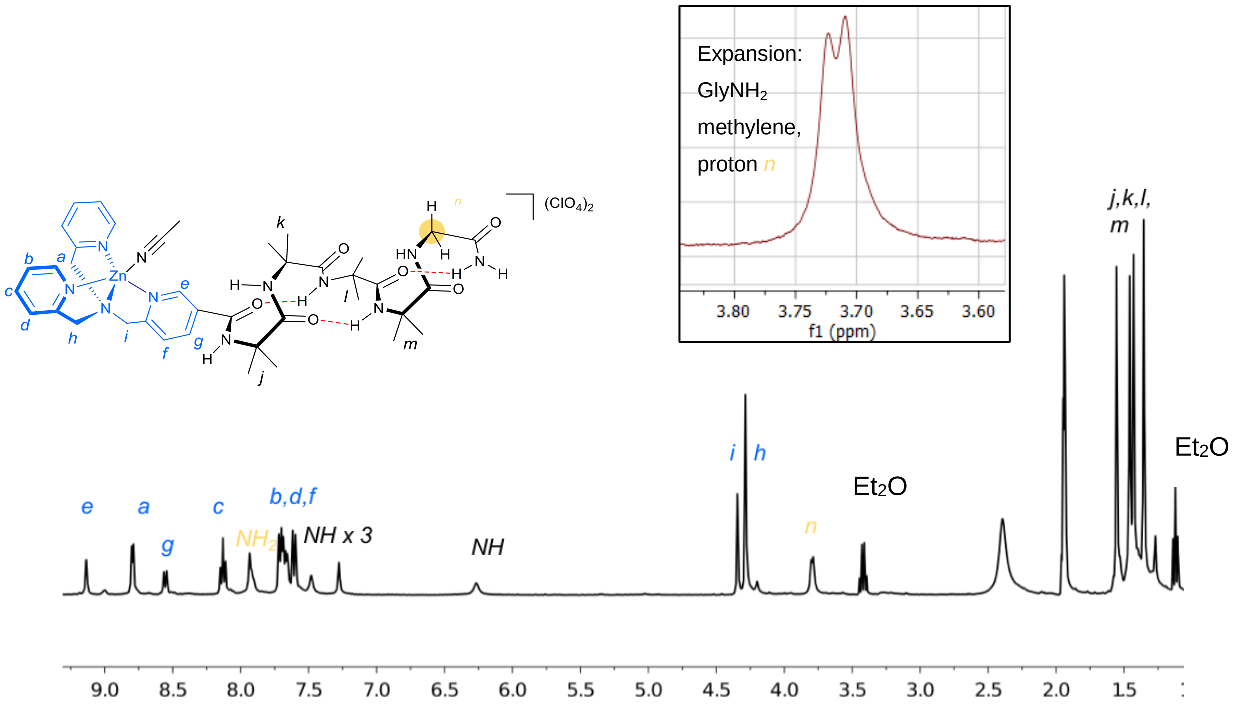

^{13}C NMR of Zn(2)·2ClO_4·Et_2O in CD_3CN

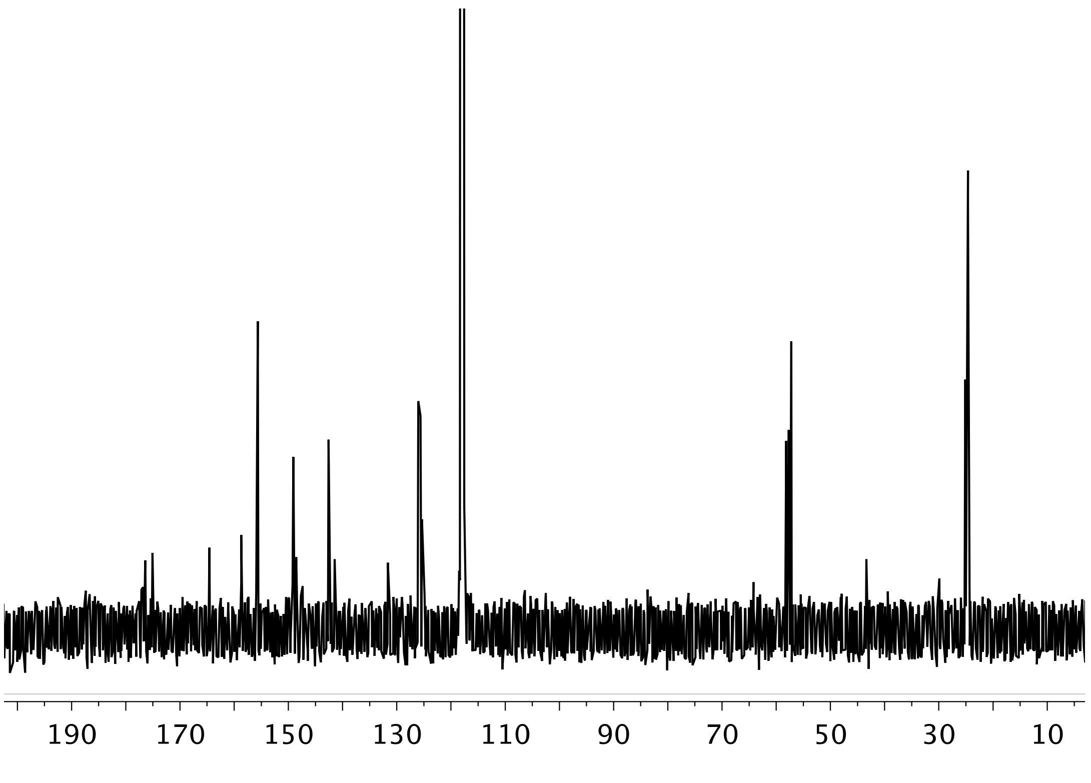

^{1}H NMR spectrum of Foldamer 3 in $CDCl_3$

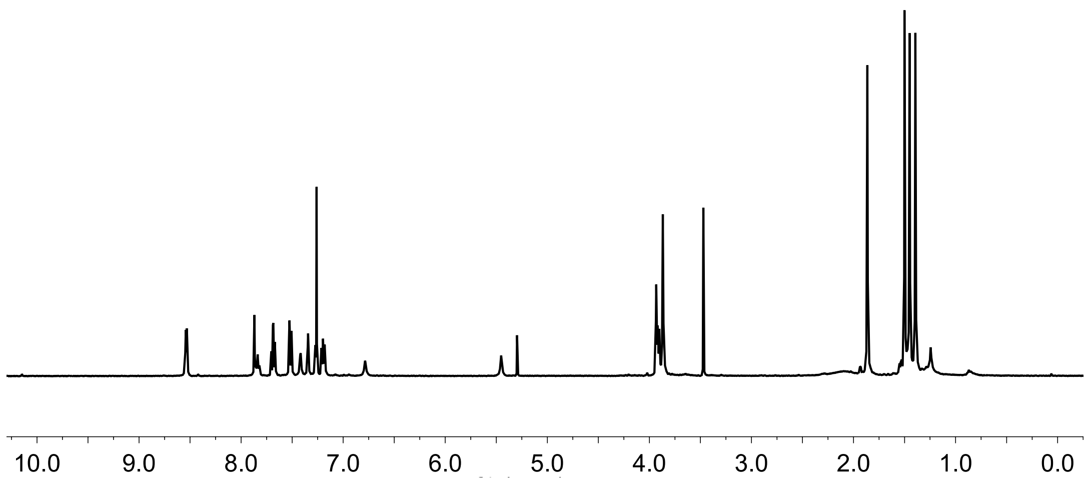

^{13}C NMR spectrum of Foldamer 3 in $CDCl_3$

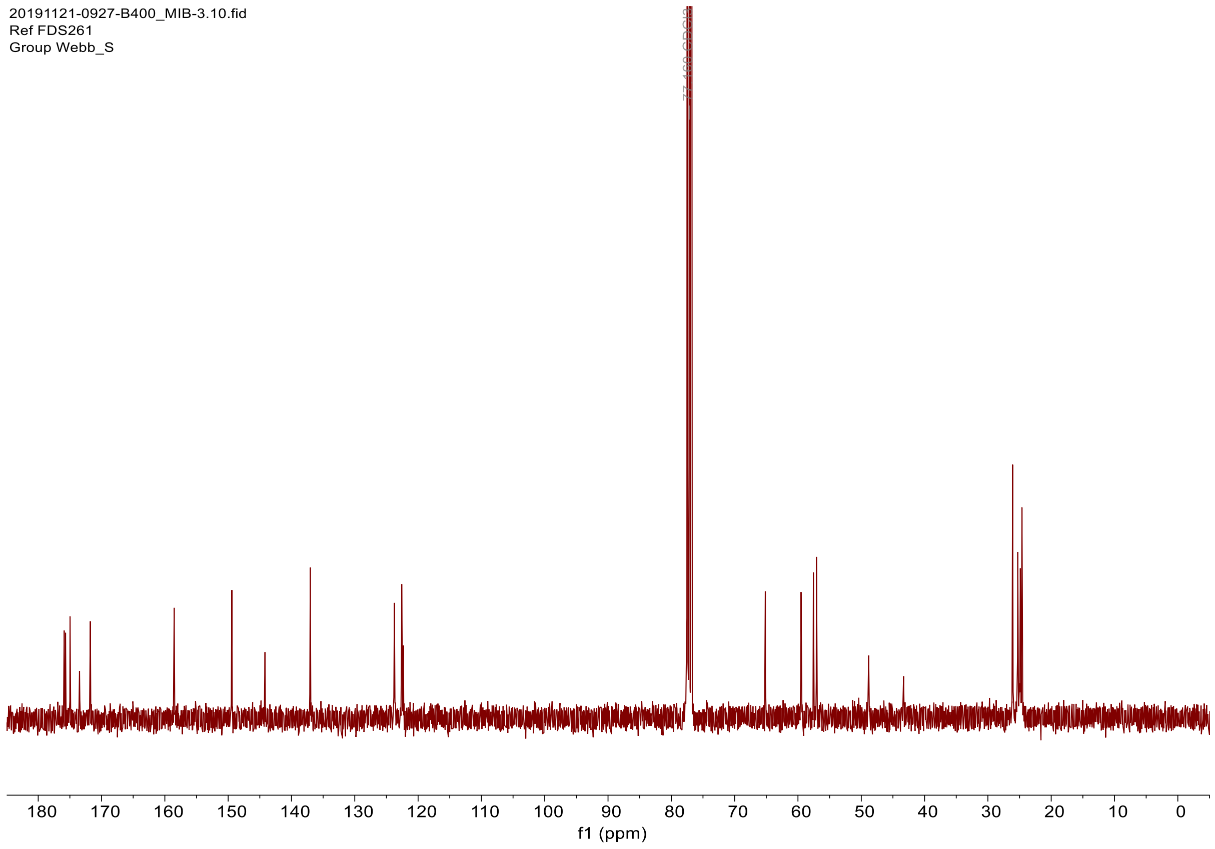

^{1}H NMR spectrum of Zn(3)·2ClO$_4$ in CD$_3$OD

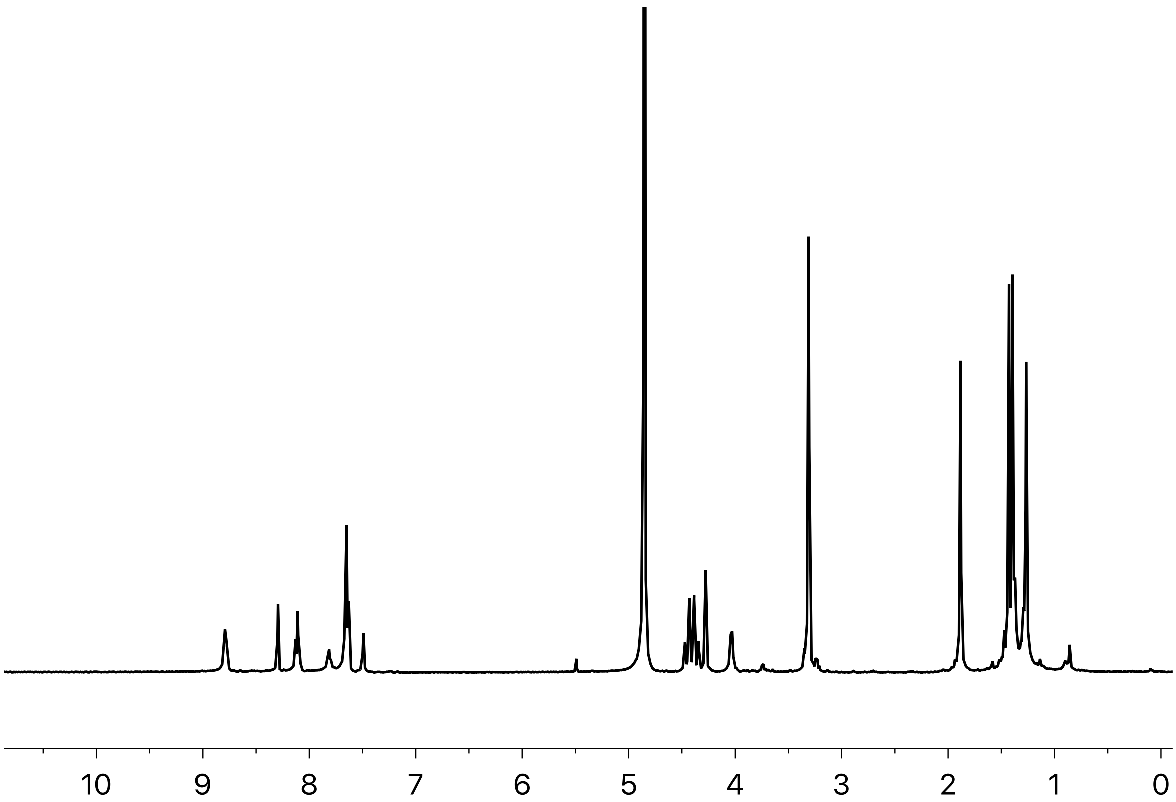

^{13}C NMR spectrum of Zn(3)·2ClO$_4$ in DMSO-d$_6$

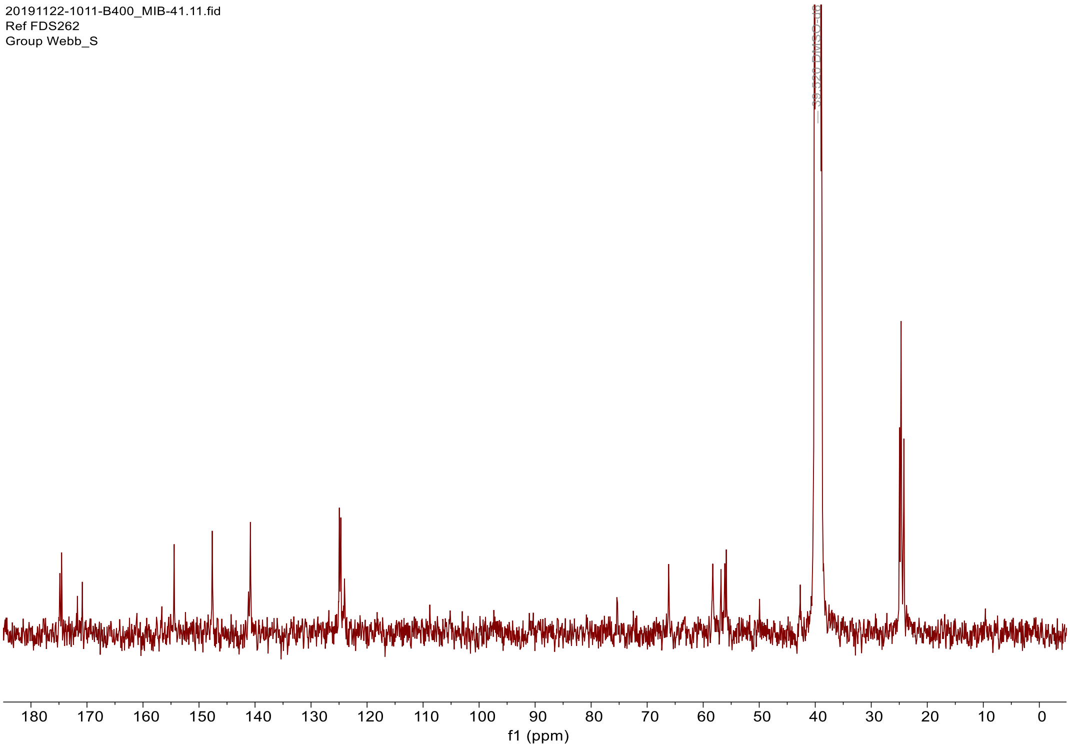

^{1}H NMR spectrum of Zn(3)·2Cl·Et$_2$O in CD$_3$OD

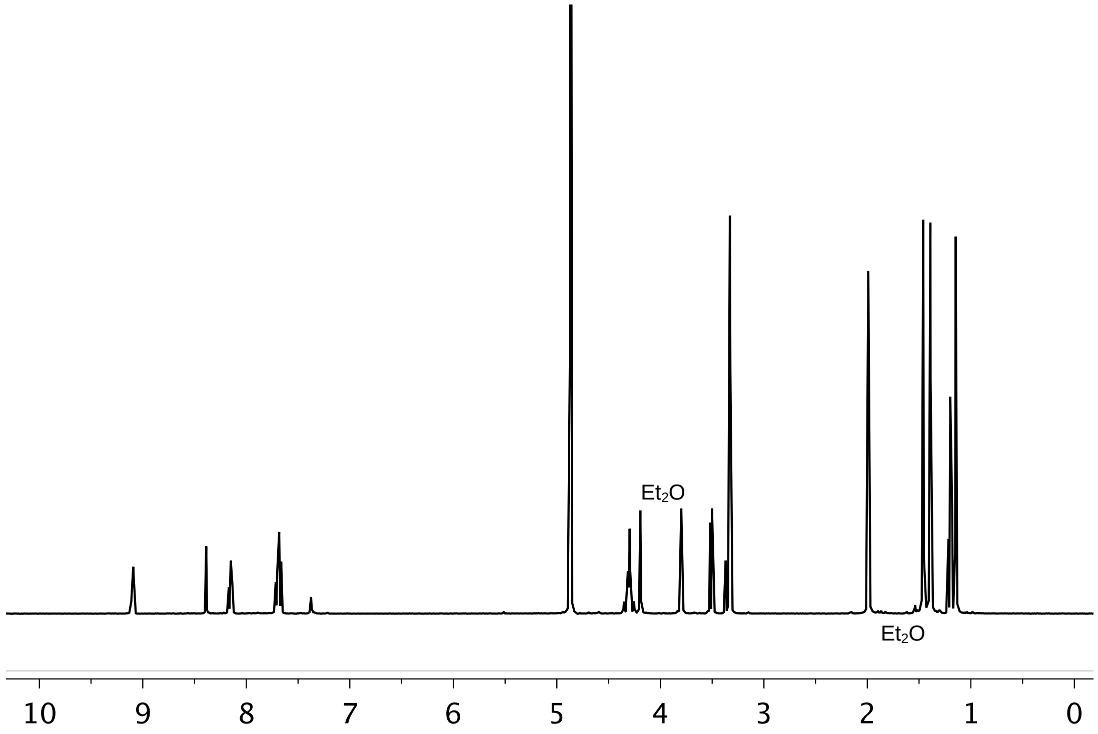

^{13}C NMR spectrum of Zn(3)·2Cl·Et$_2$O in CD$_3$OD

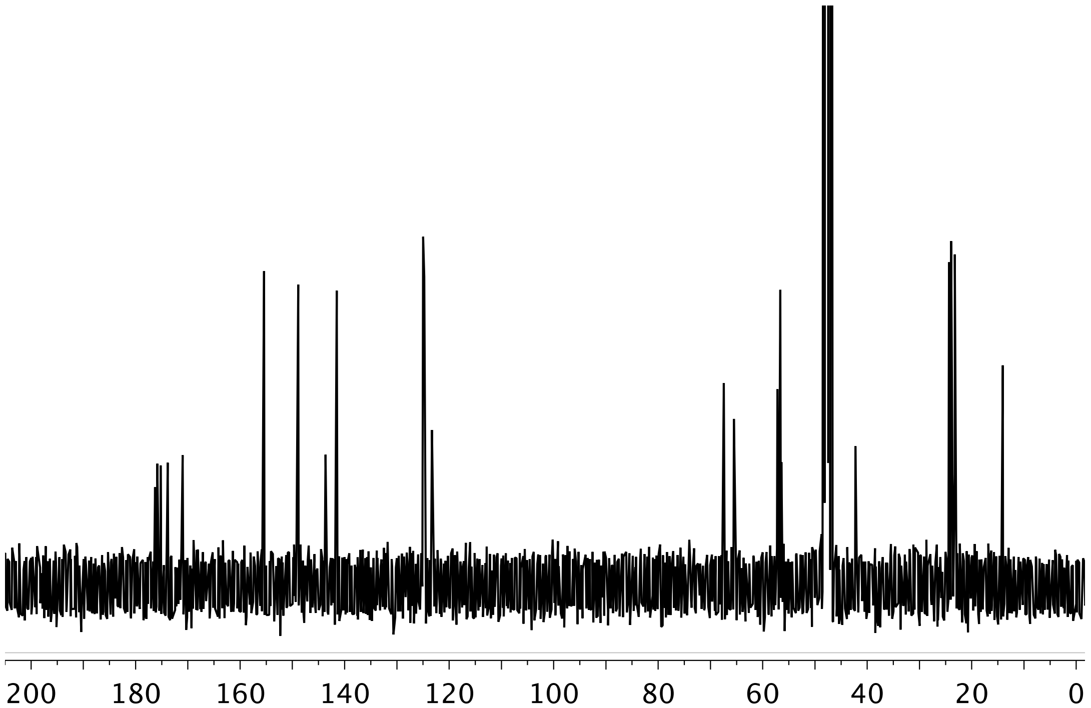

3. NMR spectroscopy studies

3.1 NMR spectroscopy experimental details

Anhydrous CD_3CN, CD_3OD, silver acetate, Boc-D-Pro and Boc-L-Pro were obtained from Sigma Aldrich. Aliquots of stock solutions were added using Gilson® pipettes or Hamilton syringes. The anisochronicity, in parts per billion (ppb), of ABX systems arising from the germinal ^{1}H nuclei of the $GlyNH_2$ diastereotopic NMR probe was given by $\upsilon_0\Delta\delta$ (ppb) = [(((*f*A1+*f*A2)/2)-((*f*B3+*f*B4)/2))*(((*f*A3+*f*A4)/2)-((*f*B1+*f*2)/2))]1/2 * 1000 where $f_{A1,A2,A3,A4,\ B1,B2,B3.,B4}$ are the observed resonant frequencies in order of the eight lines comprising of the ABX multiplet. Variable temperature NMR (VT-NMR) spectra of Zn(**3**)·2ClO_4 bound with Boc-L-Pro or *rac*-BocPro were acquired from –50 °C to 50 °C in CD_3OD (Figures **S8** and **S10** respectively). All titrations monitored by NMR spectroscopy were performed at 298 K using a Bruker AVANCE 400 MHz spectrometer.

3.2 Complexation study: titration of Zn(2)·2ClO$_4$ with Boc-D-Pro, 2,6 lutidine in CD$_3$CN

A stock solution of Boc-D-Pro (0.34 M) was made by dissolving the carboxylate (508 μmol) and 2,6-lutidine (610 μmol) in CD_3CN (1.5 mL). Zn(**2**)·2ClO_4 (5.00 mg) was dissolved in CD_3CN (500 μL) and added to an NMR tube, a spectrum was recorded for reference. For monitoring the binding of carboxylate to Zn(**2**)·2ClO_4, aliquots of Boc-D-Pro solution (15 μL = 1 eq. acid, 1.2 eq. 2,6-lutidine) were sequentially added and NMR spectra were acquired at 1, 2 ,3 and 4 eq. of Boc-D-Pro.

The extent of screw-sense control of either Zn(**2**)·2ClO_4 or Zn(**3**)·2ClO_4 by a chiral 'controller', in this case Boc-D-Pro, can be determined by NMR spectroscopy using the anisochronicity in the diastereotopic geminal protons of the glycinamide probe. This level of screw-sense control can be deduced from the anisochronicity at the glycinamide ($\Delta\delta_{gly}$); a larger splitting signifies greater control.

In the case of Zn(**2**)·2ClO_4, the addition of 1–4 equivalents of Boc-D-Pro revealed no changes in anisochronicity or chemical shifts, even for the amide NH resonances, although broadening was observed. Foldamer Zn(**2**)·2ClO_4 may be aggregating through a head-to-tail interaction, which prevents the carboxylate interacting with the Zn^{2+} metal centre. Broadening may occur due to changes in the rate of interfoldamer association.

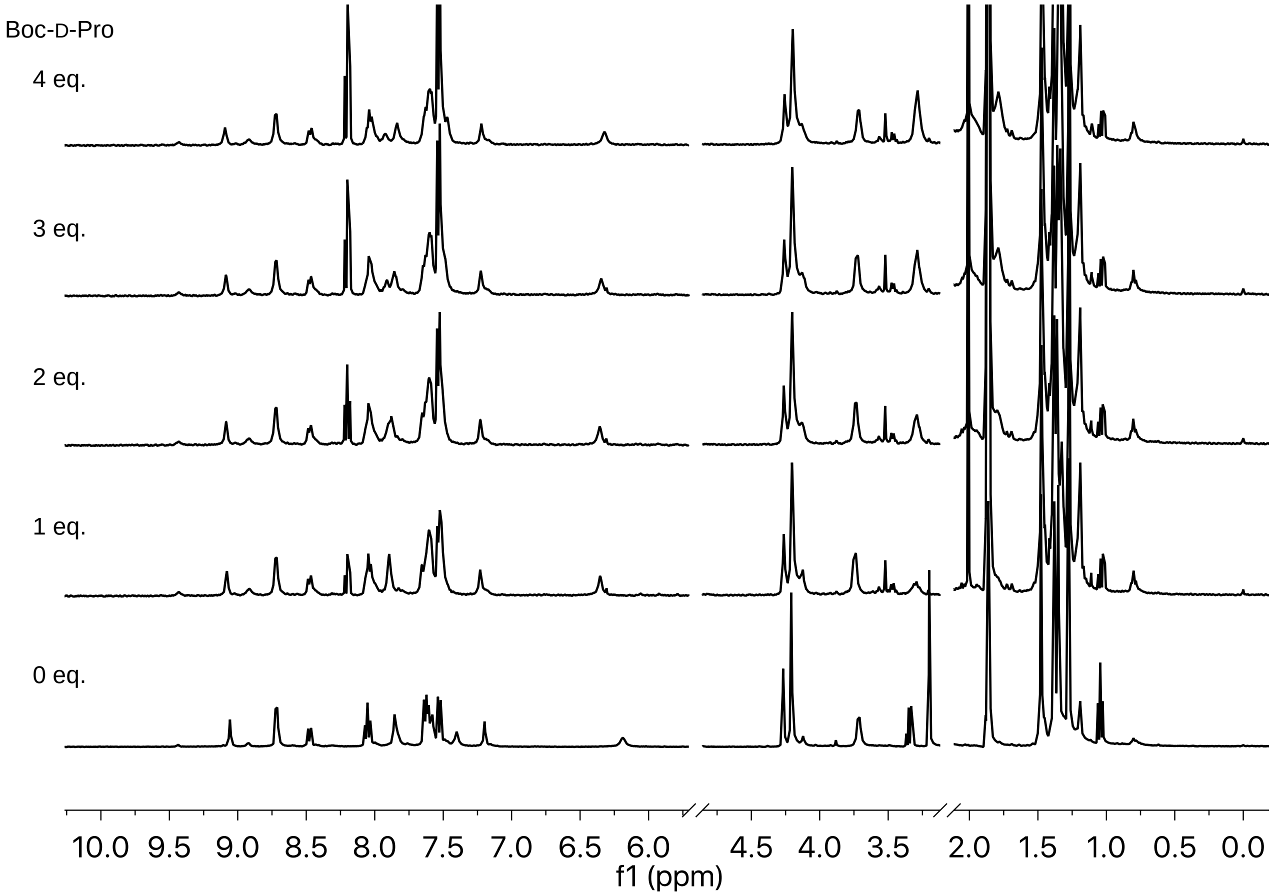

Figure S1: ^{1}H NMR titration of Boc-D-Pro (up to 4 eq.), 2,6-lutidine (up to 4.8 eq.) and Zn(**2**)·2ClO$_4$ in CD$_3$CN (400 MHz, 298 K).

3.3 Complexation study: titration of Zn(3)·$2ClO_4$ with Boc-D-Pro, 2,6 lutidine in CD_3CN

A stock solution of Boc-D-Pro was made by dissolving the carboxylate (56 μmol) and 2,6-lutidine (67 μmol) in CD_3CN (1 mL). Zn(**3**)·$2ClO_4$ (5.22 mg) was dissolved in CD_3CN (500 μL) and added to an NMR tube. For monitoring the binding of carboxylate to Zn(**3**)·$2ClO_4$, aliquots of Boc-D-Pro solution (100 μL = 1 eq. acid, 1.2 eq. 2,6-lutidine) were sequentially added and NMR spectra were acquired at 0, 1, 2 ,3 and 4 eq. of Boc-D-Pro.

The ^1H NMR spectrum after the addition of 1–4 equivalents of Boc-D-Pro to Zn(**3**)·$2ClO_4$ revealed no changes in anisochronicity or chemical shifts, alike to Zn(**2**)·$2ClO_4$. Broadening however was observed throughout the spectrum for the peaks owing to the foldamer. It is thought that Zn(**3**)·$2ClO_4$ is aggregating through a head-to-tail interaction, similar to Zn(**2**)·$2ClO_4$. This aggregation prevents the carboxylate interacting with the Zn^{2+} metal centre, thus preventing the transmission of stereochemical information to the Aib-foldamer.

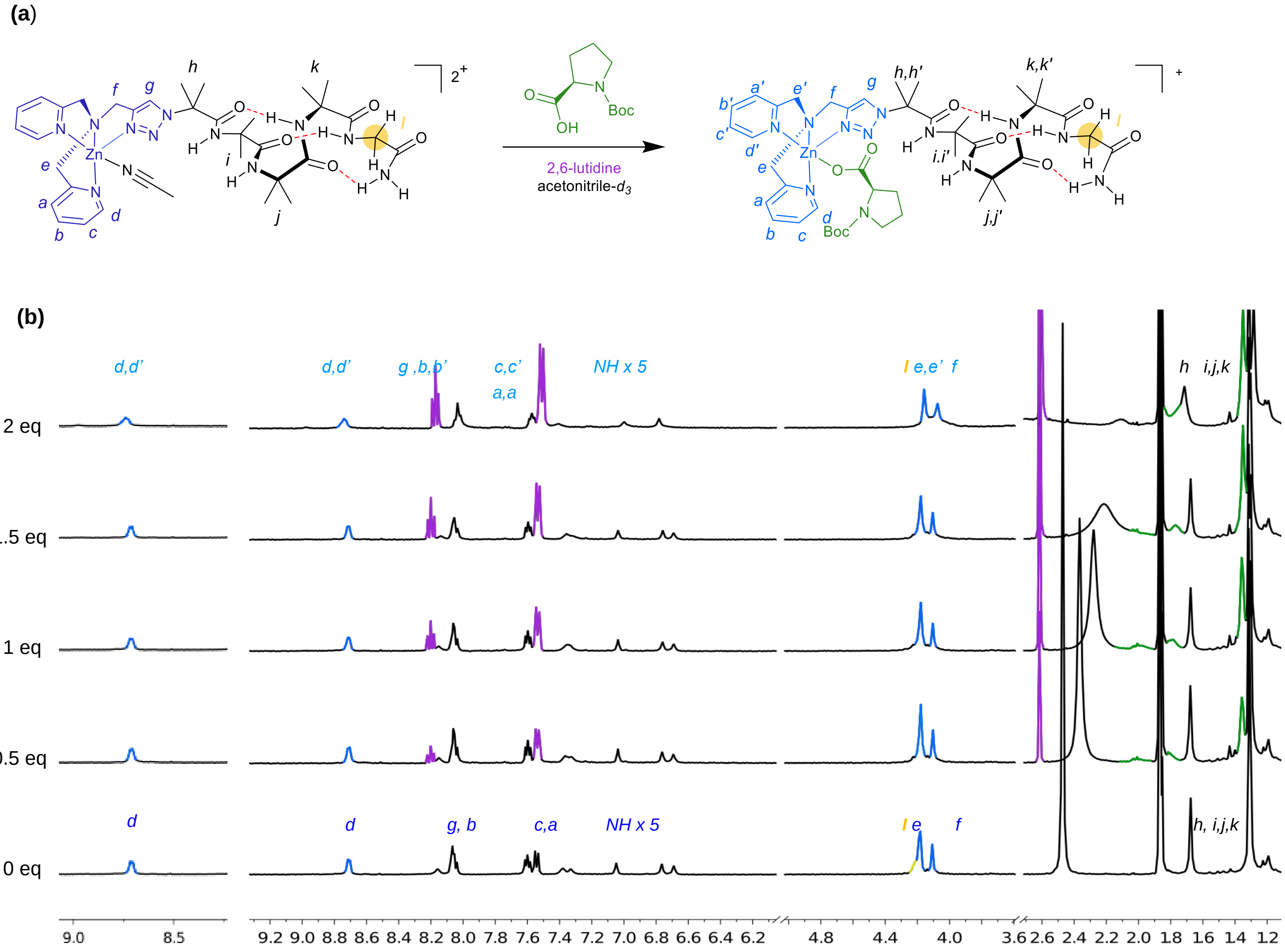

Figure S2: (a) Schematic representation of the binding of Boc-D-Pro to Zn(**3**)·2ClO$_4$; **(b)** Expansion of ^{1}H NMR spectrum displaying pyridyl protons *d* and *d'*; **(c)** ^{1}H NMR titration (400 MHz, 298 K) of Boc-D-Pro (up to 2 equiv.) with Zn(**3**)·2ClO$_4$ (0.11 M) in basic acetonitrile-d_3 (2,6-lutidine, up to 2.4 equiv.).

3.4 Complexation study: titration of Zn(3)·2ClO$_4$ with Boc-D-Pro, 2,6 lutidine in CD$_3$OD

A stock solution of Boc-D-Pro was made by dissolving the carboxylate (56 μmol) and 2,6-lutidine (57 μmol) in CD$_3$CN (1 mL). Zn(**3**)·2ClO$_4$ (5.22 mg) was dissolved in CD$_3$CN (500 μL) and added to an NMR tube. For monitoring the binding of carboxylate to Zn(**3**)·2ClO$_4$, aliquots of Boc-D-Pro solution (100 μL = 1 eq. acid, 1.2 eq. 2,6-lutidine) were sequentially added and NMR spectra were acquired at 0, 1, 2 ,3 and 4 eq. of Boc-D-Pro.

The ^{1}H NMR spectrum after the addition of 0.25, 0.5 and 0.75 equivalents of Boc-D-Pro to Zn(**3**)·2ClO$_4$ shows well-resolved and sharp resonances (Figure S3). However contrasting to Zn(**1**)·2ClO$_4$ where slow ligand exchange is apparent, fast exchange of Zn(**3**)·2ClO$_4$ with and without bound carboxylate was observed. Peak averaging for the resonances that corresponded to the two complexes were observed, this was most noticeable for the pyridyl protons, in particular proton d (Figure S3b).

The binding of the chiral 'controller' Boc-D-Pro can be observed in the pyridyl aromatic and methylene protons. On binding, the equivalent pyridyl protons become non-equivalent and anisochronicity is observed with both pyridyls now being identified individually. This was most noticeable for proton *d* of the pyridyl rings that becomes diastereotopic and spits into two separate systems (*d* and *d'*) displaying as doublets.

Unfortunately, there was no evidence of stereochemical information being relayed through the 3_{10} helix. Anisochronicity at the Aib CH$_3$ groups and the glycinamide was absent. This suggests that the triazole insulates the transmission of stereochemical information as previously seen by Boddaert *et.al* [4] and a racemic mixture of the *M* and *P* helices still exist.

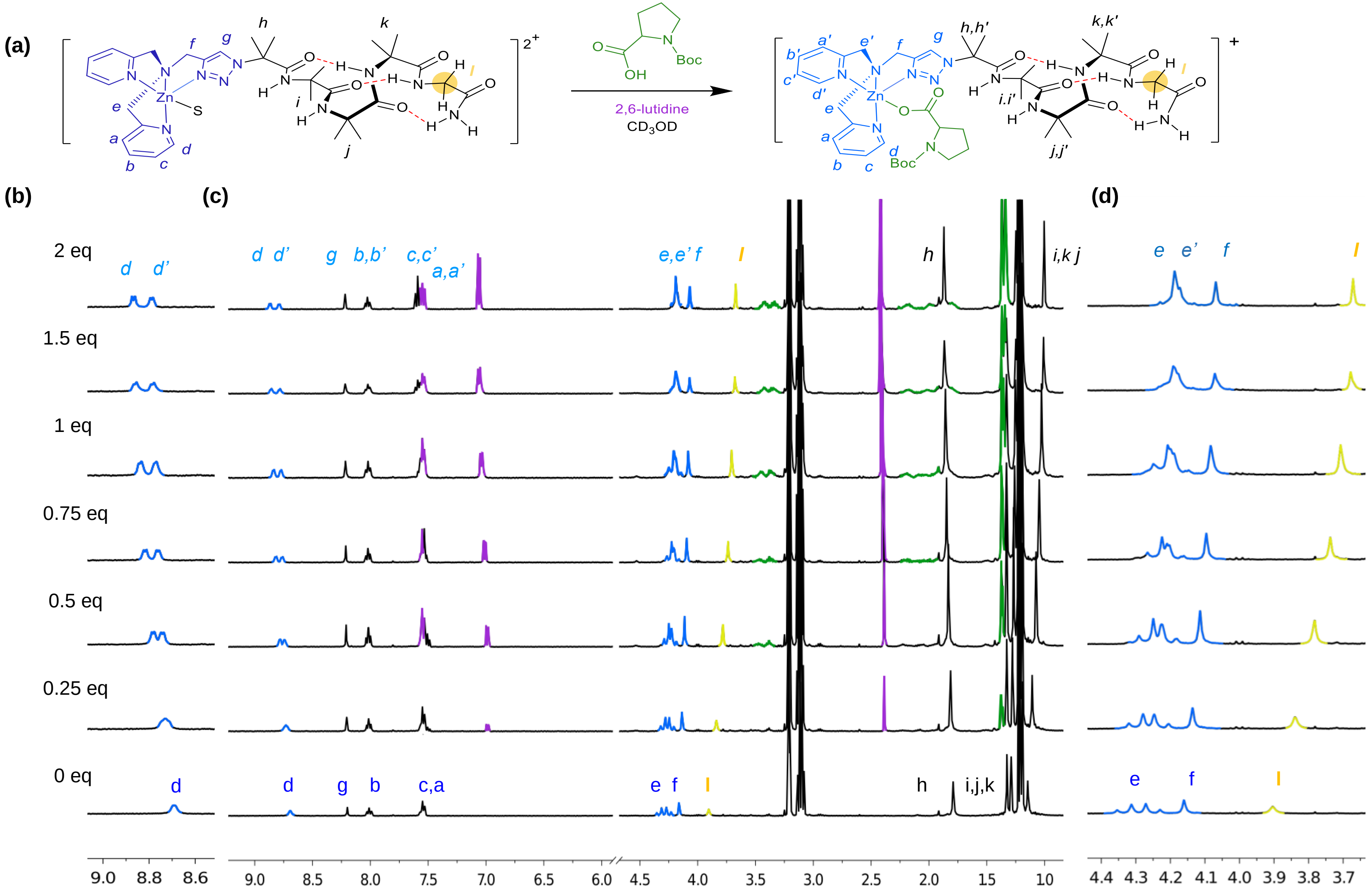

Figure S3: (a) Schematic representation of the binding of Boc-D-Pro to Zn(**3**)·2ClO4; **(b)** Expansion of ^{1}H NMR spectrum displaying pyridyl protons *d* and *d*; **(c)** ^{1}H NMR titration of Boc-D-Pro (up to 2 eq.), 2.6-lutidine (up to 2.4 eq.) with Zn(**3**) 2ClO4 (0.11 M) in CD_3OD (400 MHz, 298 K); **(d)** Expansion of ^{1}H NMR spectrum displaying Gly methylene (yellow) and pyridyl methylene residues (blue).

3.5 ^{1}H NMR spectroscopic comparison of Zn(3)·2ClO$_4$ and Zn(3)·2Cl

^{1}H NMR spectroscopy of Zn(**3**)·2ClO$_4$ and Zn(**3**)·2Cl showed that a change in counterion changed the conformation of the foldamer. The methylene protons of the glycinamide for Zn(**3**)·2Cl were shifted upfield to 3.76 ppm (from 4 ppm), back to the standard value for the GlyNH$_2$ protons. Furthermore, the methylene protons of the pyridyl and triazole arms were also shifted upfield and the AB system observed for the pyridyl arms were strongly coupled compared to Zn(**3**)·2ClO$_4$. Chemical shifts were also observed for the pyridyl arms and Aib CH$_3$ groups. These observations suggest that the chloride ion may be coordinating strongly to the Zn^{2+} metal centre.

Titration of Zn(**3**)·2Cl with Boc-D-Pro and 2,6-lutidine in methanol-*d$_4$* showed that anisochronicity was absent on the addition of Boc-D-Pro, even up to 3 equivalents. A broad singlet was observed at the α$_H$-protons of the two pyridyl arms (*d* and *d'*) suggesting that the two pyridyls are not diastereotopic. Additionally, no changes in chemical shifts or broadening were observed. These observations suggest that Boc-D-Pro does not bind with the Zn(II) metal centre because the Zn-Cl bond is too strong to be displaced by a carboxylate.

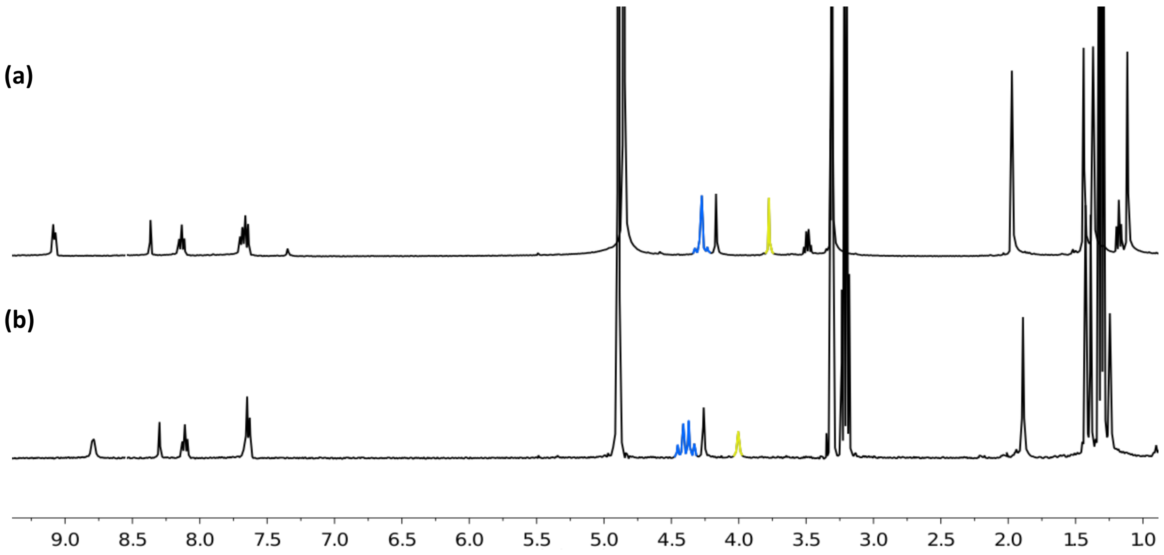

Figure S4: ^{1}H NMR spectra of (a) Zn(**3**)·2ClO$_4$ and (b) Zn(**3**).2Cl in CD$_3$OD (298 K, 400 MHz); Glycinamide methylene protons are highlighted in yellow; AB-system of PyCH$_2$ protons highlighted in blue.

3.6 ^{1}H NMR spectroscopic comparison of Zn(1)·2ClO$_4$ and Zn(2)·2ClO$_4$

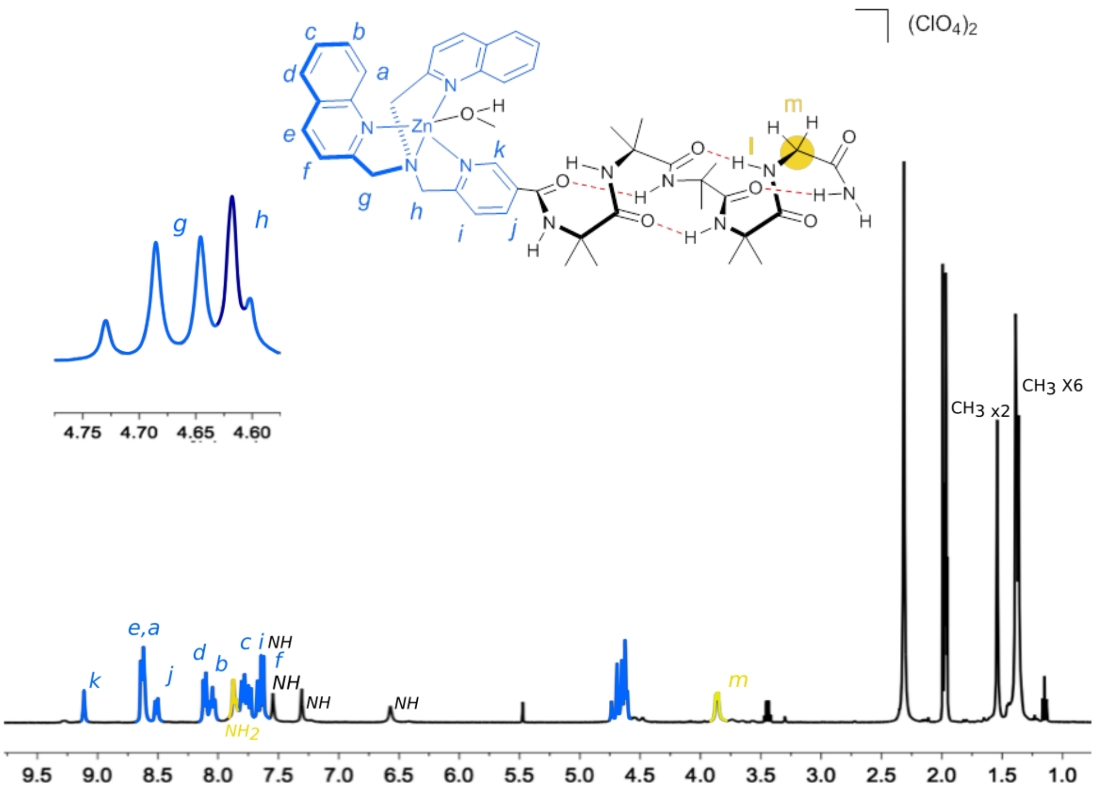

Figure S5: ^{1}H NMR spectrum of Zn(**1**).2ClO$_4$ in CD$_3$CN. The AB-system of the quinoline methylene protons is displayed in the inset.

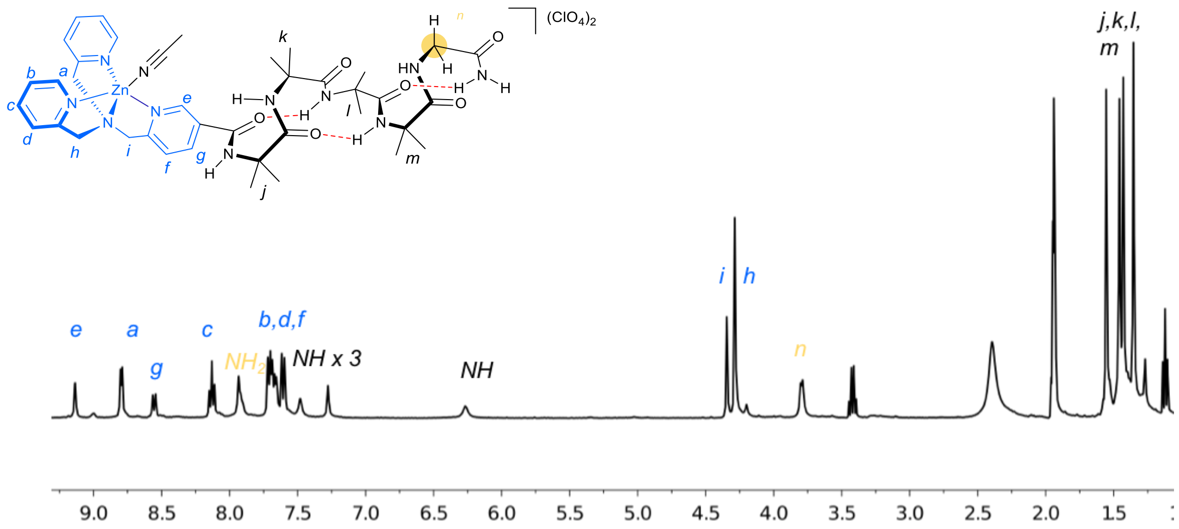

Figure S6: ^{1}H NMR spectrum of Zn(**2**)·2ClO$_4$ in CD$_3$CN

3.7 ^{1}H NMR spectroscopic comparison of Zn(3)·2ClO$_4$ in CD$_3$OD and CD$_3$CN

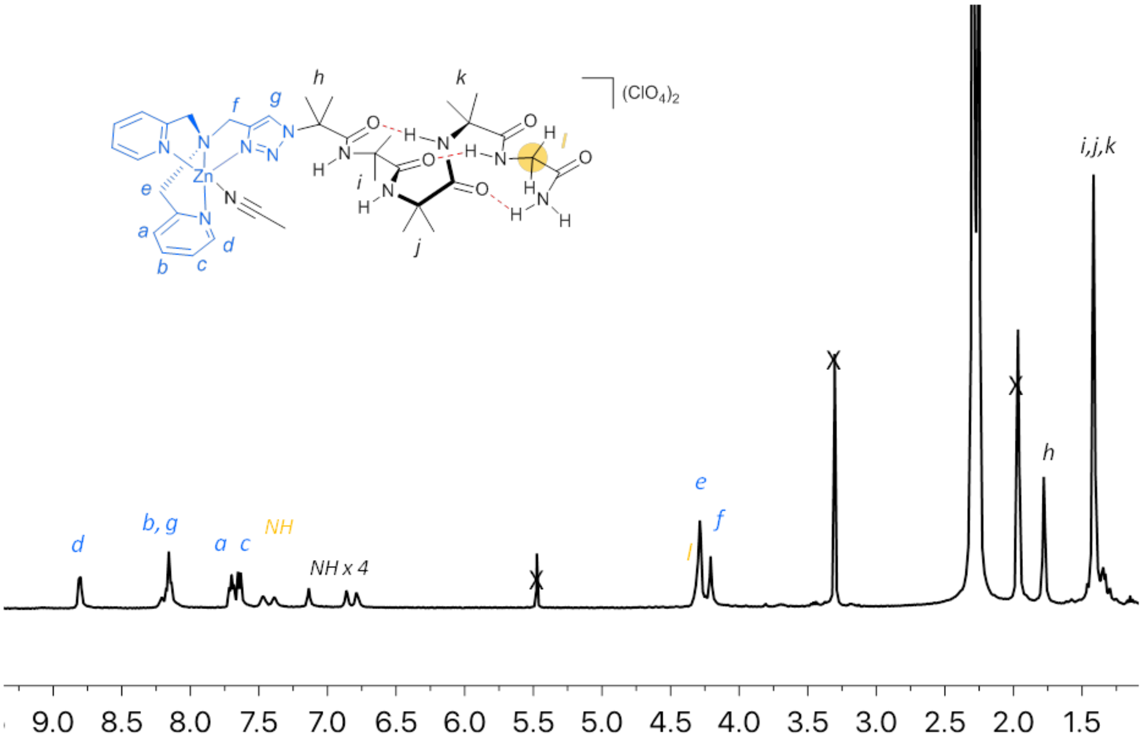

Figure S7: ^{1}H NMR spectrum of Zn(**3**)·2ClO$_4$ in CD$_3$CN

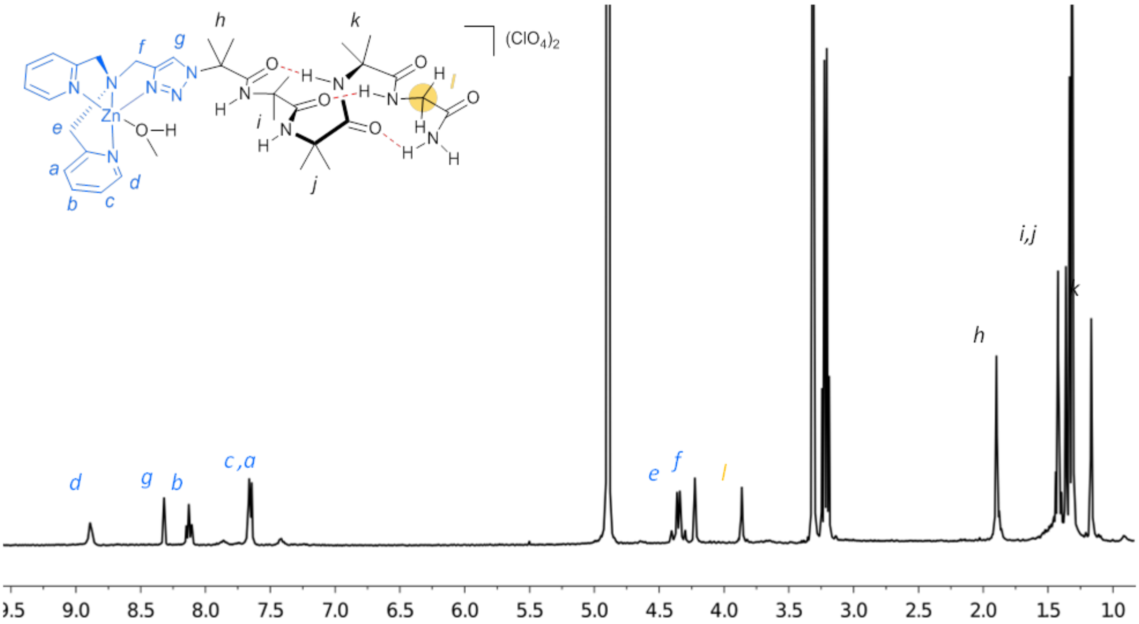

Figure S8: ^{1}H NMR spectrum of Zn(**3**)·2ClO$_4$ in CD$_3$OD

4. Variable Temperature NMR (VT-NMR) spectra of Zn(3)·2ClO₄

4.1 VT-NMR spectra of Zn(3)·2ClO₄ (either Boc-D-Pro or *rac*-BocPro bound) in CD₃OD

VT-NMR spectra of Zn(**3**)·$2ClO_4$ (Boc-L-Pro bound) and Zn(**3**)·$2ClO_4$ (*rac*-BocPro) were obtained in CD_3CN over the temperature range of 40 °C to −40 °C (Figures S4 and S6). As expected, the *cis* and *trans* rotameric forms of the coordinated BocPro were observed in both Boc-D-Pro bound and *rac*-BocPro bound Zn(**3**)·$2ClO_4$, although partially hidden by the methylene protons. Slower interchange between the *M* and *P* helical conformations of the Aib foldamer were also observed upon a decrease in temperature. Line broadening of the ABX-system for Zn(**3**)·$2ClO_4$ (*rac*-BocPro bound) ascribed to the $GlyNH_2$ protons (resonances between 3.5 and 3.7 ppm in Figure S5a) and the Aib CH_3 protons (resonances between 1.1 and 1.7 ppm in Figure S5b) were observed on a decrease in temperature from 0 °C to −40 °C. Unfortunately, decoalescence of the NMR signals was not reached within the temperature range suitable for CD_3OD.

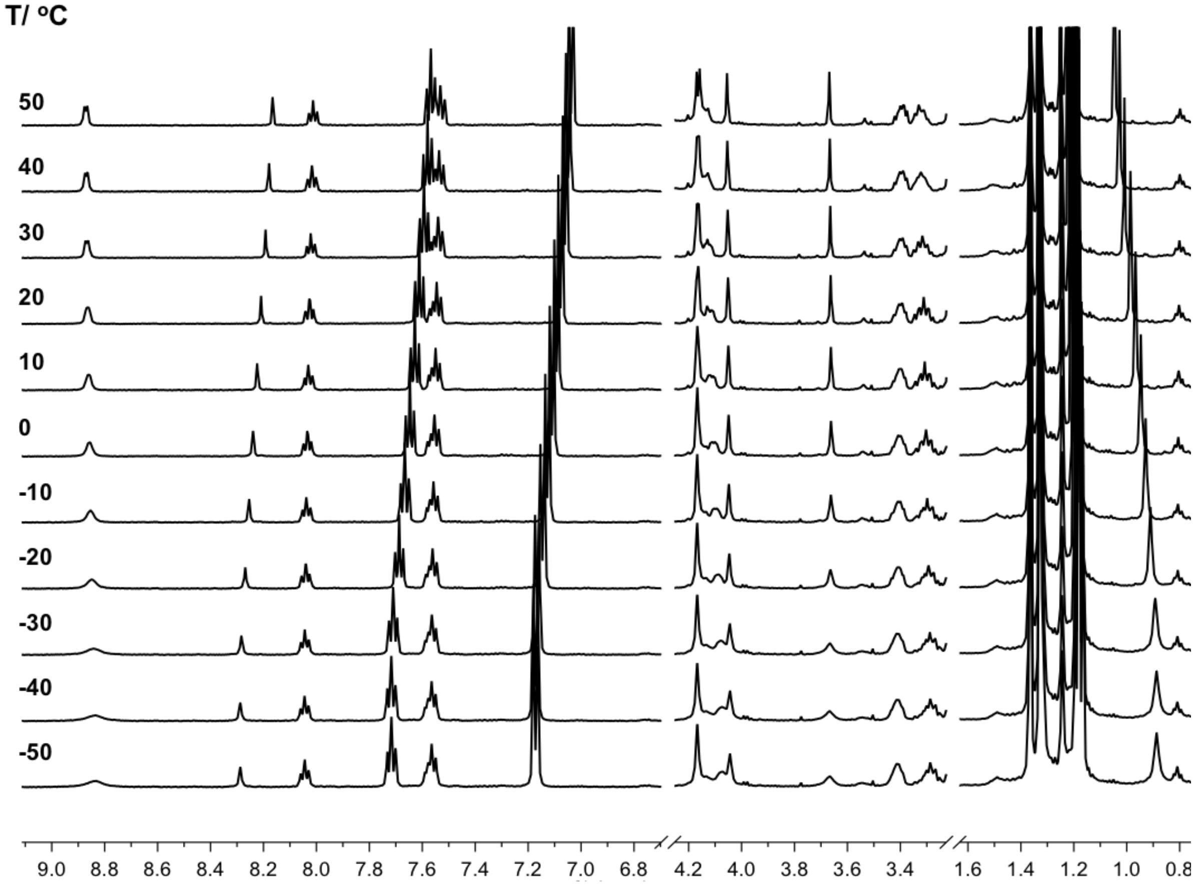

Figure S9: VT-NMR spectra of [Zn(*rac*-BocPro)(**3**)]-$2ClO_4$ from –50 °C to 50 °C in CD_3OD.

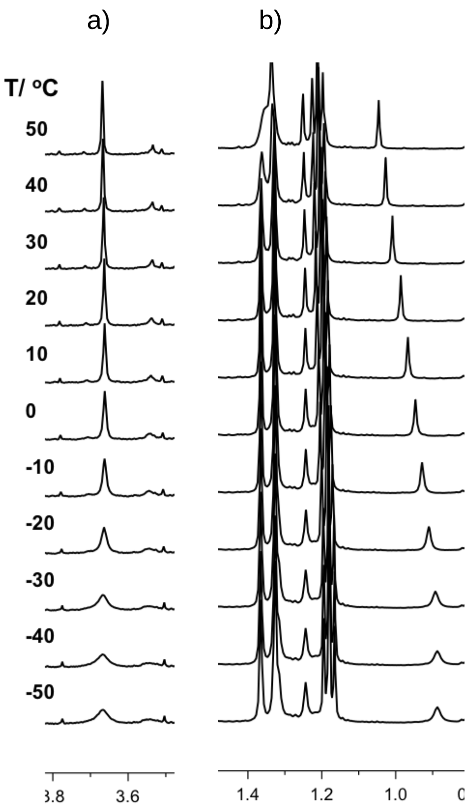

Figure S10: (a) Expansion of ^{1}H NMR spectra displaying the methylene protons of the glycinamide; (b) Expansion of ^{1}H NMR spectra displaying the Aib CH_3 peaks.

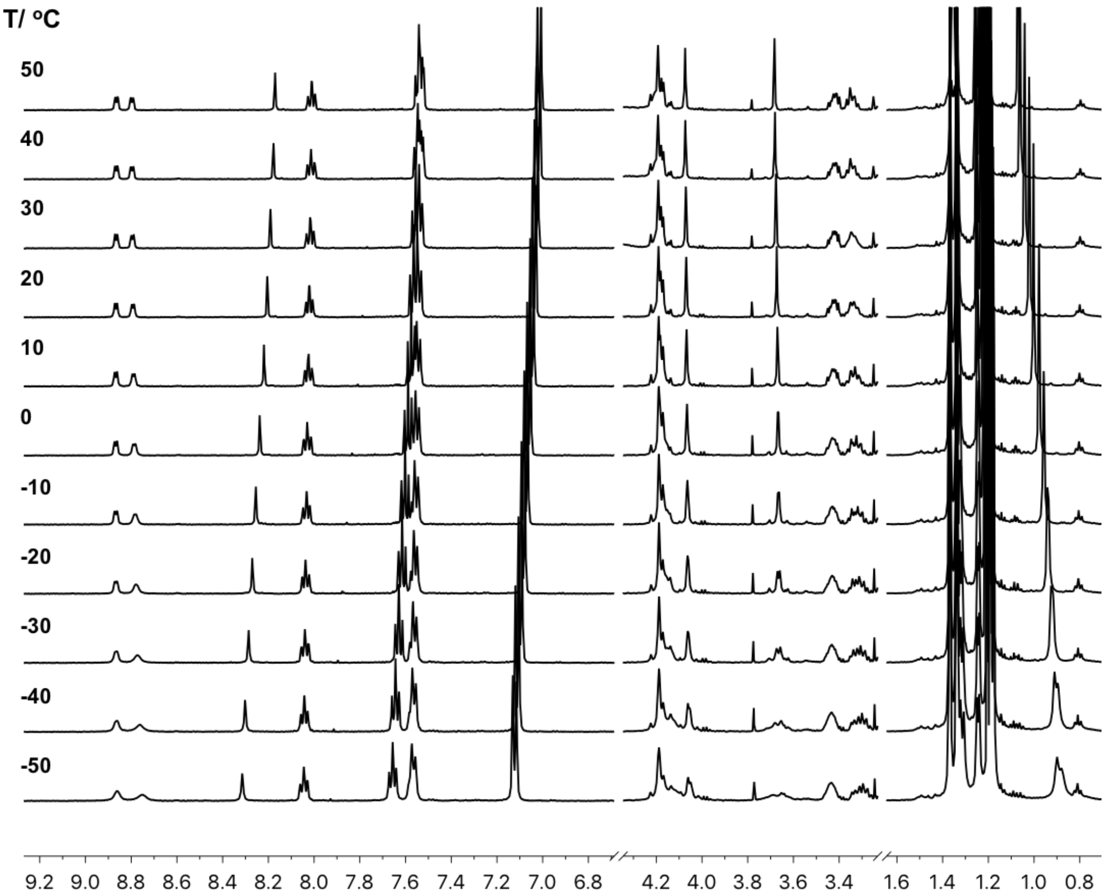

Figure S11: VT-NMR spectra of [Zn(Boc-D-Pro)(**3**)]·2ClO$_4$ from –50 °C to 50 °C in CD$_3$OD.

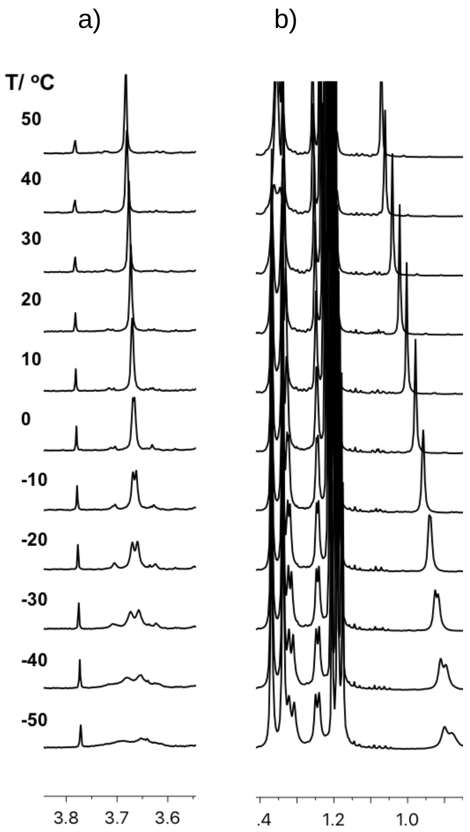

Figure S12: (a) Expansion of ^{1}H NMR spectra displaying the methylene protons of the glycinamide; **(b)** Expansion of ^{1}H NMR spectra displaying the Aib CH_3 peaks

.

5. Fitting of titration data

The change in the chemical shift of the protons at the pyridyl 2-positions of Zn(**3**)·2ClO$_4$ (0.014 M in CD$_3$OD) upon titration with 0 to 2 eq. of Boc-D-Pro and 2,6-lutidine (1.2 eq. relative to Boc-D-Pro) was measured. The resulting data was fitted to 1:1 binding isotherm using the Nelder-Mead (Simplex) algorithm in the 1:1 NMR fitter at http://app.supramolecular.org/bindfit/.[5]

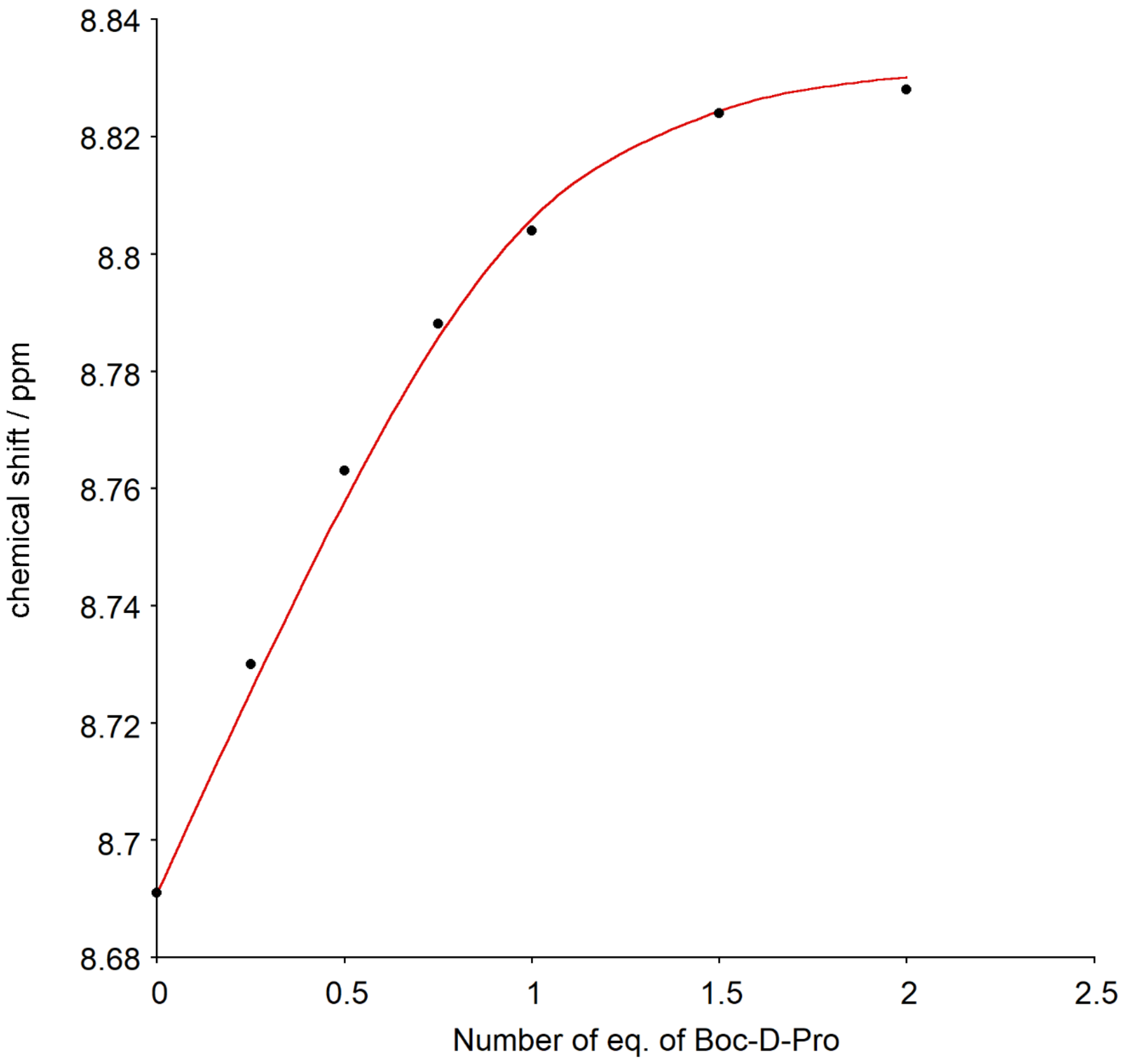

Figure S13: Plot showing the change in the chemical shift of the protons at the pyridyl 2-positions of Zn(**3**)·2ClO$_4$ (0.014 M in CD$_3$OD) upon titration with Boc-D-Pro and 2,6-lutidine (1.2 eq. relative to Boc-D-Pro). Red line shows the fit of the data a 1:1 binding isotherm with K_{app} = 1.3 × 10^3 M^{-1}.

6. Crystal data and structure refinement for Zn(2)·2ClO$_4$

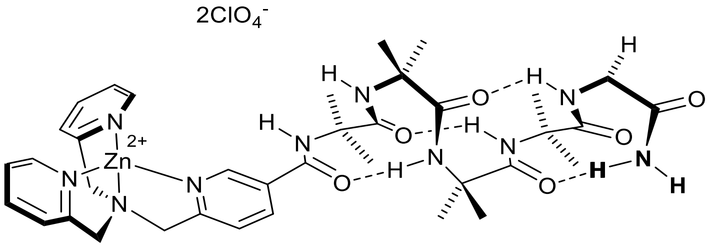

Data for Zn(**2**)·2ClO$_4$ were collected on a dual source Rigaku FR-X rotating anode diffractometer using CuK$_\alpha$ wavelength, at a temperature of 150K and reduced using CrysAlisPro 171.40.14d.[6] Absorption correction was performed using empirical methods (SCALE3 ABSPACK) based upon symmetry-equivalent reflections combined with measurements at different azimuthal angles.[6] The structure was solved using Shelxt 2014/5 and refined against all F^2 values using Shelxl 2018/1 implemented through Olex2 v1.2.10.[7,8]

Table S1. Crystallographic information for Compound Zn(**2**)·2ClO$_4$

Identification code	s5322r
Empirical formula	$C_{37}H_{56}Cl_2N_{10}O_{17}Zn$
Formula weight	1049.18
Temperature/K	150.00(10)
Crystal system	monoclinic
Space group	$P2_1/c$
a/Å	12.19330(10)
b/Å	13.05970(10)
c/Å	31.7817(3)
α/°	90
β/°	91.9190(10)
γ/°	90
Volume/Å^3	5058.11(7)
Z	4
ρ_{calc}g/cm^3	1.378
μ/mm^{-1}	2.296
F(000)	2192.0
Crystal size/mm^3	0.278 × 0.205 × 0.173
Radiation	CuKα (λ = 1.54184)
2Θ range for data collection/°	7.318 to 136.486
Index ranges	-14 ≤ h ≤ 13, -15 ≤ k ≤ 15, -38 ≤ l ≤ 33
Reflections collected	57002
Independent reflections	9226 [R_{int} = 0.0237, R_{sigma} = 0.0165]
Data/restraints/parameters	9226/1435/937
Goodness-of-fit on F^2	1.058
Final R indexes [I>=2σ (I)]	R_1 = 0.0708, wR_2 = 0.2111
Final R indexes [all data]	R_1 = 0.0729, wR_2 = 0.2131
Largest diff. peak/hole / e Å^{-3}	1.09/-0.64

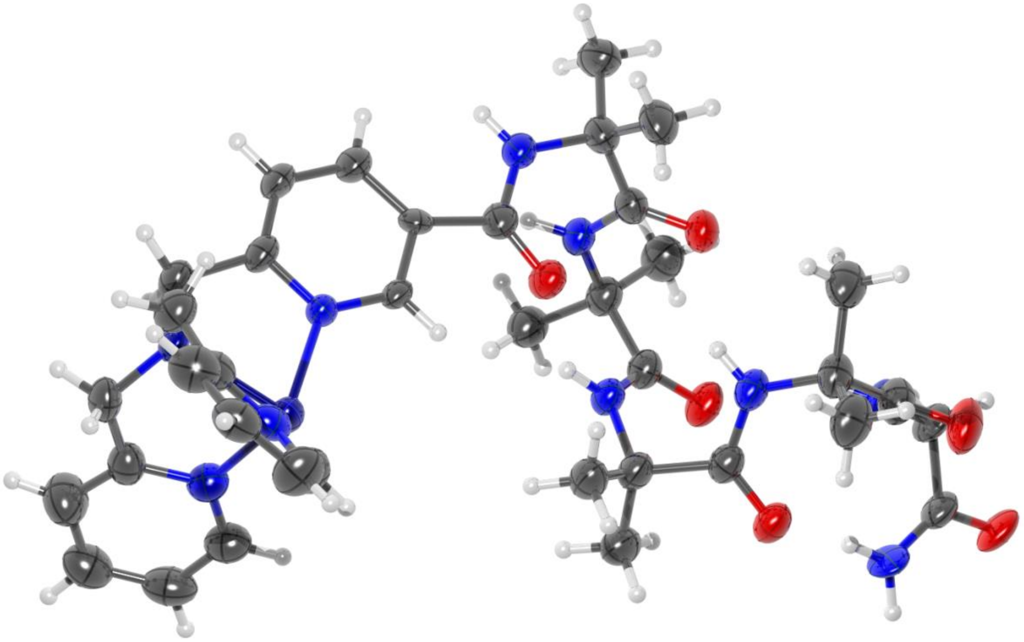

Figure S14: Representation of the asymmetric plot of Compound Zn(**2**)$\cdot$2ClO_4 in the crystal. Disorder, solvent and counter anions removed for clarity. (blue = nitrogen, white = carbon, green = fluorine, hydrogens included. Ellipsoids 50% probability). Plot produced using Olex2 and POV-ray.[8,9]

References

1 N. Yamamoto, A. K. Renfrew, B. J. Kim, N. S. Bryce, T. W. Hambley, *J. Med. Chem.*, **2012**, *55*, 11013–11021.

2 J. Clayden, A. Castellanos, J. Solà, G. A. Morris, *Angew. Chemie Int. Ed.*, **2009**, *48*, 5962–5965.

3 S. Huang, R. J. Clark and L. Zhu, *Org. Lett.*, **2007**, *9*, 4999–5002.

4 T. Boddaert, J. Solà, M. Helliwell, J. Clayden, *Chem. Commun.*, **2012**, *48*, 3397–3399.

5 (a) P. Thordarson, *Chem. Soc. Rev*., **2011**, *40*, 1305–1323. (b) D. B. Hibbert, P. Thordarson, *Chem. Commun*., **2016**, *52*, 12792–12805. (c) Fitting software accessed on http://supramolecular.org/

6 Rigaku Oxford Diffraction, **2017**, CrysAlisPro Software system, version 1.171.39.21a, Rigaku Corporation, Oxford, UK

7 (a) G. M. Sheldrick, *Acta Cryst*. **2015**, *A71*, 3–8.; (b) G. M. Sheldrick, *Acta Cryst*. **2015**, *C71*, 3–8.

8 O. V. Dolomanov, L. J. Bourhis, R. J. Gildea, J. A. K. Howard, H. Puschmann, *J. Appl. Cryst*. **2009**, *42*, 339–341.

9 Persistence of Vision Pty. Ltd., **2017**, Persistence of Vision Raytracer, Persistence of Vision Pty. Ltd., Williamstown, Victoria, Australia, http://www.povray.org/
